# Thermogenic Adipocytes Promote M2 Macrophage Polarization through CNNM4‐Mediated Mg Secretion

**DOI:** 10.1002/advs.202401140

**Published:** 2024-11-08

**Authors:** Anke Zhang, Junkun Jiang, Chuan Zhang, Houshi Xu, Wenjing Yu, Zhen‐Ning Zhang, Ling Yuan, Zhangming Lu, Yuqing Deng, Haonan Fan, Chaoyou Fang, Xiaoyu Wang, Anwen Shao, Sheng Chen, Huaming Li, Jiahua Ni, Wenhui Wang, Xiaonong Zhang, Jianmin Zhang, Bing Luan

**Affiliations:** ^1^ Department of Endocrinology Tongji Hospital Affiliated to Tongji University School of Medicine Tongji University Shanghai 200065 P. R. China; ^2^ Department of Neurosurgery Second Affiliated Hospital School of Medicine Zhejiang University Hangzhou 310009 P. R. China; ^3^ Clinical Research Center for Neurological Diseases of Zhejiang Province Hangzhou 310009 P. R. China; ^4^ Department of Neurosurgery Huashan Hospital Affiliated to Fudan University School of Medicine Fudan University Shanghai 200040 P. R. China; ^5^ Translational Medical Center for Stem Cell Therapy and Institute for Regenerative Medicine Shanghai East Hospital School of Life Sciences and Technology Tongji University Shanghai 200120 China; ^6^ School of Public Health School of Medicine Shanghai Jiaotong University Shanghai 200025 China; ^7^ Department of Neurosurgery Shanghai General Hospital School of Medicine Shanghai Jiaotong University Shanghai 200080 P. R. China; ^8^ College of Biological Science and Medical Engineering Donghua University 2999 North Renmin Road Shanghai 201620 China; ^9^ State Key Laboratory of Metal Matrix Composites School of Materials Science and Engineering Shanghai Jiao Tong University Shanghai 200240 China; ^10^ Suzhou Origin Medical Technology Co. Ltd. Suzhou 215513 China; ^11^ Brain Research Institute Zhejiang University Hangzhou 310009 P. R. China; ^12^ MOE Frontier Science Center for Brain Science and Brain‐Machine Integration Zhejiang University Hangzhou 310009 P. R. China

**Keywords:** adipose tissue thermogenesis, M2 macrophage polarization, magnesium, obesity

## Abstract

M2 macrophages promote adipose tissue thermogenesis which dissipates energy in the form of heat to combat obesity. However, the regulation of M2 macrophages by thermogenic adipocytes is unclear. Here, it is identified magnesium (Mg) as a thermogenic adipocyte‐secreted factor to promote M2 macrophage polarization. Mg transporter Cyclin and CBS domain divalent metal cation transport mediator 4 (CNNM4) induced by ADRB3‐PKA‐CREB signaling in thermogenic adipocytes during cold exposure mediates Mg efflux and Mg in turn binds to the DFG motif in mTOR to facilitate mTORC2 activation and M2 polarization in macrophages. In obesity, downregulation of CNNM4 expression inhibits Mg secretion from thermogenic adipocytes, which leads to decreased M2 macrophage polarization and thermogenesis. As a result, CNNM4 overexpression in adipocytes or Mg supplementation in adipose tissue ameliorates obesity by promoting thermogenesis. Importantly, an Mg wire implantation (AMI) approach is introduced to achieve adipose tissue‐specific long‐term Mg supplement. AMI promotes M2 macrophage polarization and thermogenesis and ameliorates obesity in mice. Taken together, a reciprocal regulation of thermogenic adipocytes and M2 macrophages important for thermogenesis is identified, and AMI is offered as a promising strategy against obesity.

## Graphical abstract

Under cold exposure, sympathetic innervation promotes adipose thermogenesis through β_3_AR to increase CNNM4 expression. Mg efflux from thermogenic adipocytes further activates mTORC2 signaling and promotes M2 macrophage polarization. Under obese conditions, lower CNNM4 expression accounts for the decreased Mg secretion and obesity. Thus, means of promoting this feedback loop by AMI can serve as valuable approaches to combat obesity.

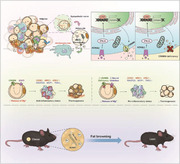

## 1 Introduction

Excessive fat accumulation in adipose tissues leads to obesity and associated metabolic diseases such as type 2 diabetes.^[^ ^1^ ^]^ Thermogenesis in brown adipose tissue (BAT) and subcutaneous white adipose tissue (scWAT) serves as a new strategy to combat against the worldwide prevalence of obesity.^[^ ^2^, ^3^, ^4^, ^5^ ^]^ The ability of these depots to dissipate energy relies largely on uncoupling protein 1 (UCP1),^[^ ^6^ ^]^ although UCP1‐independent thermogenic pathways have also been characterized in brown adipocytes.^[^ ^7^, ^8^ ^]^ Previous studies implemented cold stimuli or adrenergic signaling activation as a useful way to activate thermogenesis.^[^ ^9^, ^10^ ^]^ However, such approaches have limited applications due to various side effects on heart and other tissues.^[^ ^11^, ^12^, ^13^ ^]^ Thus, it is urgent to unveil new promising approaches to achieve relative safe and effective thermogenic activation.

Adipose tissues are composed of a variety of cells including adipocytes, immune cells and sympathetic neurons.^[^ ^14^, ^15^ ^]^ The reciprocal regulations among these different cells provide important mechanisms for thermogenic regulation. For instance, cold stimuli or adrenergic signaling activation promotes M2 macrophage polarization in adipose tissue which plays a critical role in thermogenic regulation. Numerous chemokines and secreted factors could directly or indirectly promote polarization and activation of M2 macrophage and subsequent thermogenesis.^[^ ^16^, ^17^, ^18^, ^19^ ^]^ Although the mechanism of how M2 macrophages promotes thermogenesis is poorly defined, identifying new M2 macrophage modulators may provide a promising strategy for thermogenic regulation and the treatment of obesity.

Magnesium ion (Mg) is one of the most abundant cations in human body that play important roles in metabolic regulation. Mg deficiency is a great risk factor for obesity which leads to type 2 diabetes and metabolic syndrome.^[^ ^20^, ^21^ ^]^ Thus, Mg supplement may be a potential therapeutic strategy for the treatment of obesity. However, Mg absorption is impaired in individuals with BMI>35 kg m^−1[^ ^22^, ^23^, ^24^ ^]^ and several clinical trials reveal that the benefits of oral Mg supplementation are controversial.^[^ ^21^, ^25^ ^]^ Thus, a more precise and long‐term release approach will shed light on the clinical use of Mg against obesity and type 2 diabetes. Meanwhile, it is still unclear how Mg can exert its beneficial effect on metabolism. Here, we identified Mg as a thermogenic adipocyte‐secreted factor to induce M2 macrophage polarization and thermogenesis in adipose tissue. In obesity, the downregulation of Cyclin and CBS domain divalent metal cation transport mediator 4 (CNNM4), a transporter mediating Mg efflux, decreased Mg secretion from thermogenic adipocytes and led to decreased M2 macrophage polarization and energy expenditure. Moreover, we employed an Mg wire implantation (AMI) system for long‐term controlled release of Mg in scWAT to ameliorate obesity and this approach could serve as a potential new therapeutic strategy against obesity and metabolic diseases.

## 2 Result

### 2.1 Thermogenic Adipocytes Promote M2 Macrophage Polarization in Adipose tissue

**Figure 1:**
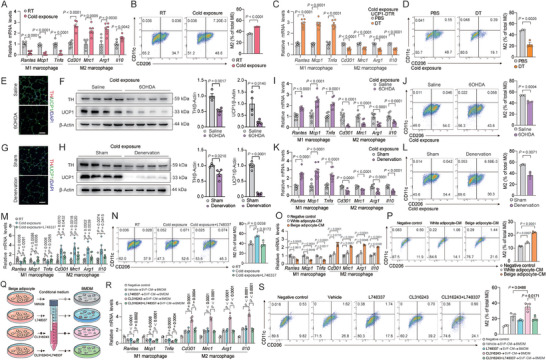
Thermogenic adipocytes promote M2 macropage polarization in adipose tissue. A) Representative M1 and M2 macrophage marker gene expression (*n* = 6) and B) flow cytometric plots and quantification demonstrate the numbers of M2 macrophages (CD206^+^/CD11c^−^) (*n* = 3) in scWAT from C57BL/6 mice under room temperature (25 °C) or cold exposure (4 °C) for 2 d; C) representative M1 and M2 macrophage marker gene expression (*n* = 6) and D) flow cytometric plots and quantification demonstrate the numbers of M2 macrophages (CD206^+^/CD11c^−^) (*n* = 3) in scWAT from UCP1‐DTR mice injected with DT (200 ng/mice/d) or PBS; E,G) representative TH and UCP1 immunostaining in scWAT, F,H) UCP1 and TH protein expression, I,K) M1 and M2 macrophage marker gene expression (*n* = 8), and J,L) representative flow cytometric plots and quantification demonstrate the numbers of M2 macrophages (CD206^+^/CD11C^−^) (*n* = 3) in scWAT of mice with 6‐OHDA injection (E, F, I, and J) or surgical transection of sympathetic neuron fibers (G, H, K, and L) under cold exposure for 2 d. Scale bar, 50 µm; M) representative M1 and M2 macrophage marker gene expression (*n* = 6) and N) flow cytometric plots and quantification demonstrate the numbers of M2 macrophages (CD206^+^/CD11c^−^) (*n* = 3) in scWAT of mice locally injected with ADRB3 selective antagonist L748337 (5 mg kg^−1^) or PBS under cold exposure; O) representative M1 and M2 macrophage marker gene expression (*n* = 4) and P) flow cytometric plots and quantification demonstrate the numbers of M2 macrophages (CD206^+^/CD11c^−^) (*n* = 3) in BMDMs stimulated with conditioned medium (CM) of white adipocyte or beige adipocyte for 48 h; Q) scheme illustrating experimental setup; R) representative M1 and M2 macrophage marker gene expression (*n* = 4) and S) flow cytometric plots and quantification demonstrate the numbers of M2 macrophages (CD206^+^/CD11c^−^) (*n* = 3) in BMDMs stimulated with CM from unstimulated beige adipocytes (vehicle) or CL316243, L748337, CL316243+L748337 treated beige adipocytes for 48 h. Data were expressed as means ± SEM. (A)–(D), (F), and (H)–(L) were calculated by unpaired two‐tailed Student's *t*‐test; (M)–(S) were calculated by two‐way ANOVA followed with Bonferroni's multiple comparison test.

Cold exposure has been shown to promote M2 macrophage polarization in subcutaneous white adipose tissue (scWAT) and brown adipose tissue (BAT) (**Figures** **1**A,B and S1A,B, Supporting Information),^[^ ^26^ ^]^ although the underling mechanisms are poorly characterized. To explore whether cold‐induced thermogenic adipocytes contribute to M2 macrophage polarization, we eliminated scWAT and BAT thermogenic adipocytes using diphtheria toxin (DT)‐injected UCP1‐DTR mice (UCP1‐Cre, DTR‐STOP^fl/fl^) under cold exposure as previously reported.^[^ ^27^ ^]^ Interestingly, we found that M2 macrophage marker genes expression was downregulated while M1 macrophage marker genes expression was upregulated in scWAT and BAT of DT‐injected compared to PBS‐injected UCP1‐DTR mice when subjected to cold exposure for 2 d (Figure 1C and Figure S1C, Supporting Information). Consistently, flow cytometry analysis of the stromal vascular fraction (SVF) from scWAT and BAT showed decreased composition of M2 macrophages (CD11c‐low/CD206‐high) in DT‐injected UCP1‐DTR mice under cold exposure (Figure 1D and Figure S1D, Supporting Information). Cold induces sympathetic innervation and the release of catecholamine stimulates thermogenic adipocyte formation and activation.^[^ ^28^ ^]^ To further confirm the effects of thermogenic adipocytes on M2 macrophage polarization, we used local injection of 6‐hydroxidopamine (6‐OHDA) as well as surgical denervation strategy to ablate the sympathetic fibers in scWAT or BAT.^[^ ^27^ ^]^ As a result, the expression of sympathetic neuron marker gene tyrosine hydroxylase (TH) and thermogenic marker gene UCP1 was decreased (Figure 1E–H and Figure S1E–H, Supporting Information). At the meantime, M2 macrophage polarization as detected by marker genes expression and flow cytometry analysis was also inhibited after 6‐OHDA treatment or surgical transection when mice were subjected to cold exposure for 2 d (Figure 1I–L and Figure S1I–L, Supporting Information). β_3_ adrenergic receptor (ADRB3) on adipocytes mediates sympathetic neuron‐induced thermogenesis. Indeed, local injection of ADRB3 selective antagonist L748337 in scWAT also decreased the M2 macrophage polarization under cold exposure (Figure 1M,N). Moreover, as is shown in Figure S1M–P (Supporting Information), under the thermoneutral condition when thermogenic adipocytes were deactivated, M2 macrophage polarization marker genes were not significantly changed after local administration of 6‐hydroxidopamine (6‐OHDA) as well as surgical denervation, indicating thermogenic adipocytes activation was critical for M2 macrophage polarization.

To further prove this concept, we differentiated SVF of scWAT to primary beige adipocytes and stimulated the cells with ADRB3 agonist CL316243 or ADRB3 selective antagonist L748337. We collected the conditioned medium (CM) from these cells and applied them to primary bone marrow derived macrophages (BMDMs). We found that M2 polarization of BMDM as detected by both marker genes expression and flow cytometry analysis was increased by beige adipocytes‐derived CM compared to control CM (Figure 1O,P). Furthermore, medium from CL316243‐stimulated beige adipocytes further increased M2 macrophage polarization, which was abolished when beige adipocytes were pretreated with ADRB3 selective antagonist L748337 (Figure 1Q–S). We also repeated the same experiments with CM from SVF‐differentiated white adipocytes and similar but to a less extent effect was observed (Figure 1O,P and Figure S2A,B, Supporting Information). Importantly, we excluded the direct effects of CL316243 or L748337 on BMDMs (Figure S2C, Supporting Information). These results suggested that thermogenic adipocytes promoted M2 macrophage polarization through secreted factors.

### 2.2 Thermogenic Adipocyte‐Secreted Mg Promotes M2 Polarization via mTORC2

**Figure 2:**
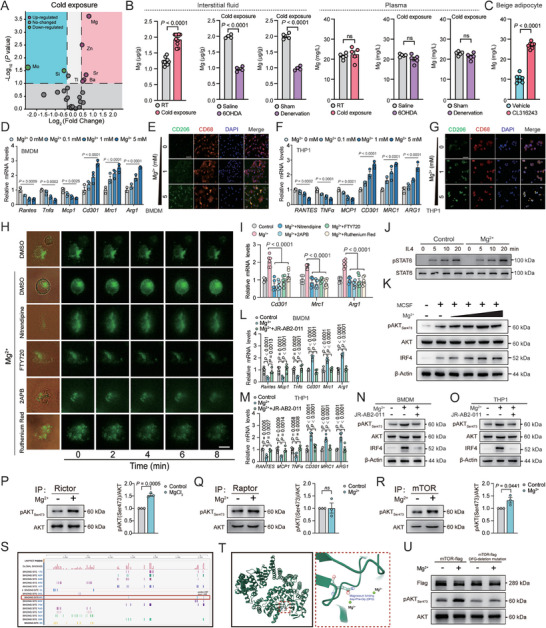
Thermogenic adipocyte‐secreted Mg promotes M2 polarization via mTORC2. A) Volcano plot of ions altered in scWAT‐derived interstitial fluid from C57BL/6 mice under room temperature (25 °C) or cold exposure (4 °C) (*n* = 4); B) Mg levels in scWAT‐derived interstitial fluid and plasma from mice with cold exposure or 6‐OHDA injection or surgical transection of sympathetic neuron fibers under cold exposure (*n* = 4–8); C) Mg levels in the medium from beige adipocytes treated with CL316243 (2 µg mL^−1^) (*n* = 7); D,F) representative M1 and M2 macrophage marker gene expression (*n* = 3) and E,G) staining of CD206 and CD68 in BMDMs or THP1 cells after stimulation with indicated concentration of MgCl_2_. Scale bar, 50 µm; H) representative staining showing the influx of Mg into BMDMs after stimulation with MgCl_2_ (5 × 10^−3^ m) and nitredipine, FTY720, 2APB, or Ruthentic Red. Scale bar, 20 µm; I) representative M2 macrophage marker gene expression (*Cd301, Mrc1*, and *Arg1*) in BMDMs after stimulation with MgCl_2_ (5 × 10^−3^ m) and nitredipine, FTY720, 2APB, or Ruthentic Red (*n* = 6); J) representative immunoblots showing effects of Mg treatment on IL4‐induced STAT6 phosphorylation at indicated time in BMDMs; K) representative immunoblots showing effects of Mg treatment on MCSF‐induced mTORC2 mediated AKT phosphorylation and IRF4 expression at 30 min in BMDMs. (concentrations of Mg^2+^: 0.1, 1, and 5 × 10^−3^ m); L,M) representative M1 and M2 macrophage marker gene expression (n = 4) and N,O) immunoblots showing effects of Mg treatment on MCSF‐induced mTORC2 mediated AKT phosphorylation and IRF4 expression in BMDMs or THP1 cells stimulated with MgCl_2_ (5 × 10^−3^ m) and mTORC2 selective inhibitor JR‐AB2‐011; P–R) examination of mTORC2 activity IPed with Rictor, Raptor, and mTOR through in vitro kinase assay after Mg stimulation on BMDMs (*n* = 3); S,T) potential Mg binding sites and Mg binding pocket (Asp‐Phe‐Gly) in serine/threonine‐protein kinase mTOR in BMDMs; U) mTORC2 activity with WT and Mg‐binding mutant of mTOR through in vitro kinase assay after Mg stimulation on BMDMs. Data were expressed as means ± SEM. (B), (C), and (P)–(R) were calculated by unpaired two‐tailed Student's *t*‐test; (D) and (F) were calculated by one‐way ANOVA; (I), (L), and (M) were calculated by two‐way ANOVA followed with Bonferroni's multiple comparison test.

Ion imbalance has been linked with obesity and inflammation which prompted us to investigate whether ions might mediate the crosstalk between thermogenic adipocytes and M2 macrophage polarization, although other protein or lipid factors might also mediate this crosstalk.^[^ ^23^, ^29^ ^]^ We collected the interstitial fluid of scWAT from room temperature (RT, 25 °C) and cold (4 °C) exposed mice to ion mass spectrum analysis and found that Mg was the most upregulated ion under cold exposure (**Figure** **2**A,B). Sympathetic denervation by 6‐OHDA treatment or surgical transection abrogated the increase of the interstitial Mg levels under cold exposure, while plasma levels of Mg remained unchanged (Figure 2B). We also collected the interstitial fluid of BAT and similar results were obtained (Figure S3, Supporting Information). Meanwhile, Mg levels were increased in the medium from CL316243‐stimulated beige adipocytes (Figure 2C). We next used MgCl_2_ solution to directly test the role of Mg in M2 macrophage polarization. Indeed, Mg treatment on BMDMs or human monocytic cell line THP1 cells greatly increased M2 macrophage marker genes expression but inhibited M1 macrophage marker genes expression in a dose‐dependent manner (Figure 2D–G).

According to previous study, through CNNM4, Mg^2+^ is exchanged for sodium,^[^ ^30^ ^]^ and a deficiency leads to a disturbed Ca^2+^ balance.^[^ ^31^ ^]^ Therefore, we further explored the effects of these ions on macrophage polarization. We treated BMDMs with an equivalent concentration of Ca^2+^, Na^+^, and Cl^−^ (CaCl_2_: 5 × 10^−3^ m, NaCl: 10 × 10^−3^ m, or MgCl_2_: 5 × 10^−3^ m) and revealed that Mg^2+^ but not Ca^2+^, Na^+^, or Cl^−^ promoted M2 macrophages polarization (Figure S4, Supporting Information). Moreover, Mg treatment did not influence the expression of thermogenic genes and mitochondrial biogenic genes when applied directly to primary beige adipocytes (Figure S5, Supporting Information), indicating that Mg targeted on macrophages instead of adipocytes. Mg is transported into cells mainly by transient receptor potential (TRP) cation channels including TRPM7, TRPM6, and MAGT1.^[^ ^32^, ^33^ ^]^ Indeed, when BMDMs were pretreated with Nitrendipine (50 × 10^−6^ m, MAGT1 inhibitor), 2APB (100 × 10^−6^ m, TRPM7 inhibitor), FTY720 (3 × 10^−6^ m, TRPM7 inhibitor), or Ruthenium red (50 × 10^−6^ m, TRPM6 inhibitor), Mg influx into BMDM was largely suppressed (Figure 2H). As a result, the promotive effect of Mg on M2 macrophage polarization was also abrogated (Figure 2I). Similar results were obtained when TRPM7 was knocked down in BMDM (Figure S6, Supporting Information). These results clearly showed that thermogenic adipocyte‐secreted Mg promoted M2 macrophage polarization.

M2 macrophage polarization is mainly maintained by IL‐4‐STAT6 or macrophage colony stimulating factor (MCSF)‐mTORC2 signaling.^[^ ^34^, ^35^ ^]^ To assess the mechanism of Mg on M2 macrophage polarization, we first examined the influence of Mg on these two pathways in BMDMs. Surprisingly, Mg did not affect IL4‐induced STAT6 phosphorylation, while it greatly promoted MCSF‐induced mTORC2 activation as shown by increased phosphorylation of mTORC2 downstream targets AKT (Ser473) in a dose‐dependent manner in BMDM (Figure 2J,K).

It has been reported that MCSF stimulates mTORC2 activation and mTORC2 in turn promotes M2 macrophage polarization through IRF4‐mediated increase in metabolic reprograming.^[^ ^34^, ^36^ ^]^ Indeed, increased IRF4 expression was observed after Mg treatment in a dose‐dependent manner in BMDMs (Figure 2K). Furthermore, we collected the tissue‐resident macrophages (through magnet‐activated cell sorting [MACS]) and mature adipocytes from scWAT after local injection of MgCl_2_ or PBS. The phosphorylation of AKT (Ser473) and IRF4 expression were increased in macrophages but not in mature adipocytes after MgCl_2_ injection (Figure S7A,B, Supporting Information). Importantly, the promotive effect of Mg on M2 macrophage marker genes and IRF4 expression as well as phosphorylation of mTORC2 downstream targets was abrogated by mTORC2 selective antagonist JR‐AB2‐011 (Figure 2L–O), suggesting that Mg promoted M2 macrophage polarization through mTORC2.

We then performed in vitro kinase assay to directly test the effect of Mg on mTORC2 activation. Mg treatment increased phosphorylation of purified AKT at Ser473 by mTORC2 (IP by Rictor) but not mTORC1 (IP by Raptor) (Figure 2P–R). To determine the mechanism of Mg on the activation of mTORC2 complex, we investigated the crystal structure of serine/threonine‐protein kinase mTOR and found an active Mg binding motif (Asp‐Phe‐Gly, DFG) in mTOR (Figure 2S,T). Indeed, deletion mutation of DFG abrogated the promotive effect of Mg on mTORC2 activity (Figure 2U). These results indicated that Mg promoted M2 macrophage polarization via direct binding to mTOR.

### 2.3 CNNM4 Mediates Mg Release from Thermogenic Adipocytes

**Figure 3:**
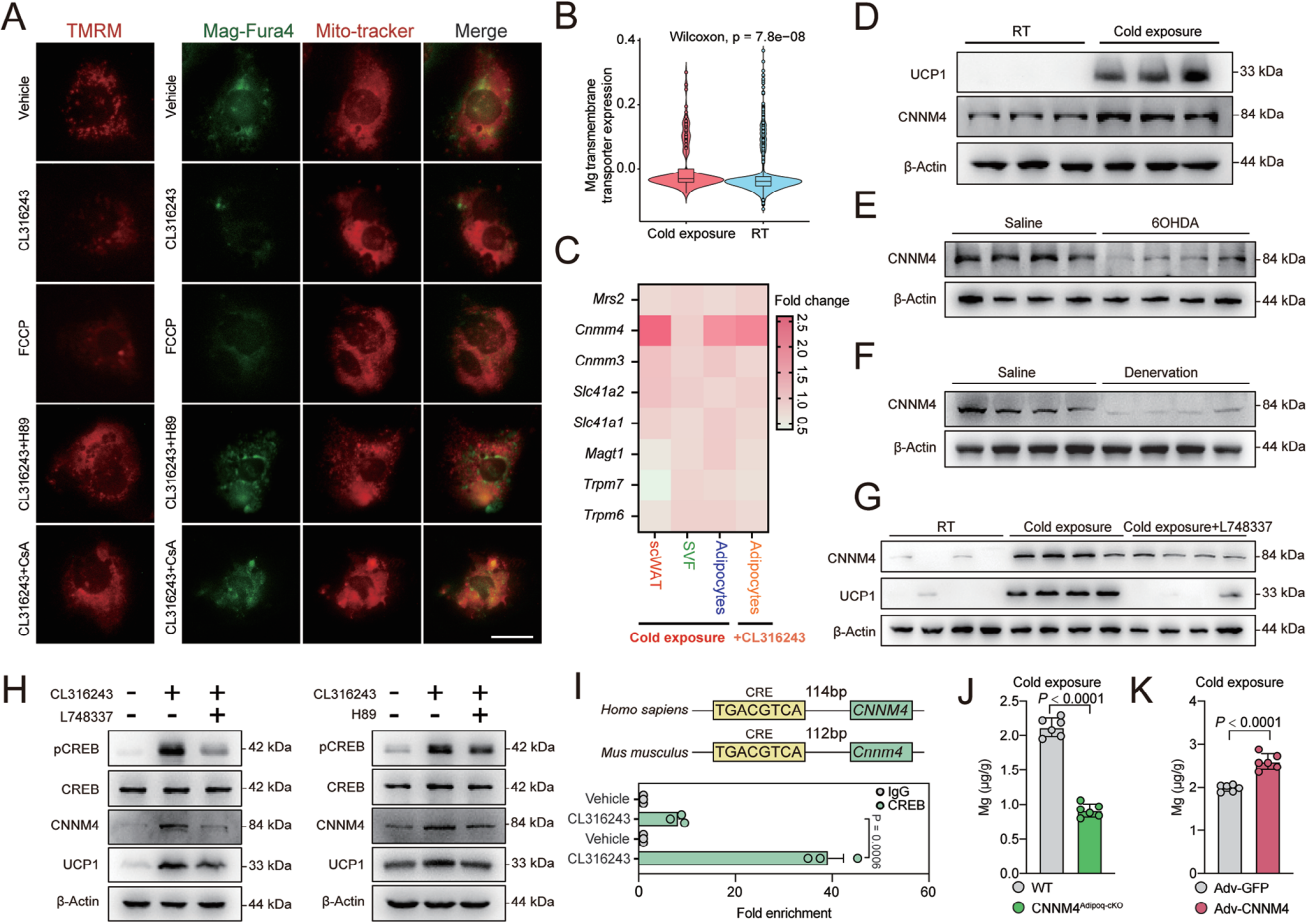
CNNM4 mediates Mg release from thermogenic adipocytes. A) Representative staining of TMRM, MitoTracker, and Mag‐Fluo‐4 in beige adipocytes treated with indicated compounds (CL316243 2 µg mL^−1^, H89 10 × 10^−6^ m, FCCP 5 × 10^−6^ m, CsA 100 × 10^−9^ m). Scale bar, 20 µm; B) Mg transmembrane transporter expression in adipocytes from mice under cold exposure or RT through single cell sequencing from literature; C) fold change of the Mg transporters gene expression (*Mrs2, Cnnm4, Cnnm3, Slc41a1, Slc41a2, Magt1, Trpm6*, and *Trpm7*) in scWAT, SVF, or adipocytes under cold exposure or CL316243 stimulation; D) UCP1 and CNNM4 protein expression in scWAT from mice under room temperature (25 °C) or cold exposure (4 °C); E–G) UCP1 and CNNM4 protein expression in scWAT from mice with 6‐OHDA injection, surgical transection of sympathetic neuron fibers, or local injection with L748337 under cold exposure; H) UCP1 and CNNM4 protein expression and CREB phosphorylation in primary beige adipocytes treated with CL316243 (2 µg mL^−1^) together with L748337 (4 × 10^−6^ m) or H89 (10 × 10^−6^ m); I) ChIP‐qPCR analysis of CREB binding to CNNM4 promoter in primary beige adipocytes treated with CL316243. Result was normalized to input control values and represented as fold enrichment relative to the anti‐rabbit IgG control (*n* = 3); J) Mg levels in scWAT‐derived interstitial fluid from CNNM4^Adipoq‐cKO^ or WT mice (*n* = 6); K) Mg levels in scWAT‐derived interstitial fluid from C57BL/6 mice with local injection of Adv‐GFP or Adv‐CNNM4 (*n* = 6). Data were expressed as means ± SEM. (B), (C), (J), and (K) were calculated by unpaired two‐tailed Student's *t*‐test; (I) were calculated by two‐way ANOVA followed with Bonferroni's multiple comparison test.

As thermogenic adipocytes are enriched with mitochondria where intracellular Mg is predominantly stored in,^[^ ^6^ ^]^ we hypothesized that ADRB3 activation might induce Mg release from mitochondria. It has been demonstrated that PKA induces depolarization of mitochondrial membrane to release Ca and Zn from the mitochondria^[^ ^27^, ^37^, ^38^, ^39^ ^]^ and we proposed that a similar mechanism may also apply to Mg. Through cationic tetramethylrhodamine methylester (TMRM) staining, we confirmed that CL316243 or Carbonyl cyanide 4 (trifluoomethoxy) phenylhydrazone (FCCP) induced the depolarization of mitochondrial membrane in beige adipocytes. Pretreatment with PKA inhibitor (H89) remarkedly blocked CL316243‐induced mitochondrial membrane depolarization, indicating the dependence on PKA signaling. Similarly, the co‐localization of the Mg label with MitoTracker staining was decreased after stimulation of CL316243 or FCCP, suggesting the release of Mg from mitochondria, while pretreatment with H89 abrogated this effect (**Figure** **3**A). Meanwhile, blocking MPTP, a voltage‐dependent multiple‐component protein which plays an important role in mitochondrial depolarization with cyclosporine A (CsA) resulted in decreased mitochondria membrane depolarization and Mg release from mitochondria (Figure 3A).

Mitochondrial membrane depolarization mediated the Mg release into the cytoplasm, while ion transporters are responsible for Mg efflux from the cytoplasm to the extracellular space. Indeed, we noted that a series of Mg transmembrane transporter expression (*Cldn16, Nipa1, Nipal1, Slc41a1, Cnnm4, Nipal4, Zdhhc13, Cnnm2, Nipal3, Mrs2, Nipal2, Tusc3, Nipa2, Magt1, Slc41a2*, and *Mmgt1*) were increased in adipocytes from mice under cold exposure comparing to those in RT using single‐cell sequencing data from literature^[^ ^40^ ^]^ (Figure 3B). Therefore, we screened a series of Mg transporter (*Mrs2, Cnnm3, Cnnm4, Trpm6, Trpm7, Magt1, Slc41a1*, and *Slc41a2*) in scWAT from mice under cold exposure and found that *Cnnm4* expression was significantly increased (Figure 3C and Figure S8A, Supporting Information).

We were able to confirm the increased expression of CNNM4 after cold exposure and the decreased CNNM4 expression after sympathetic denervation or administration with ADRB3 antagonist L748337 under cold exposure (Figure 3D–G) in scWAT. In addition, we isolated mature adipocytes and SVF, respectively, from scWAT and found that the increased expression of CNNM4 was limited in mature adipocytes (Figure S8B,C, Supporting Information). While ADRB3 agonist CL316243 stimulation increased the expression of CNNM4 in primary beige adipocytes (Figure 3H and Figure S8D, Supporting Information), pretreatment with ADRB3 antagonist (L748337) or PKA inhibitor (H89) abrogated the increase (Figure 3H). Meanwhile, pretreatment of primary beige adipocytes with Cycloheximide (CHX) to inhibit new protein synthesis also blocked CL316243‐stimulated CNNM4 expression, indicating that CNNM4 is a primary transcriptional target of ADRB3 signaling (Figure S9A,B, Supporting Information). Indeed, we noted a cAMP response element (CRE) sequence (TGACGTCA) in the promoter region of *Cnnm4* and confirmed that CREB bond to this region by ChIP experiment, which was further increased upon CL316243 stimulation (Figure 3I). To further verify the binding of CREB to CNNM4 promoter, we constructed a luciferase plasmid containing the CRE region of CNNM4 promoter. PKA agonist forskolin stimulation resulted in an increase in luciferase activity. However, when CREB binding site was mutated (TGACGTCA to ACTGCAGT) in the luciferase reporter construct, forskolin was unable to stimulate its activity (Figure S9C, Supporting Information).

CNNM4 serves as Mg transporter involving in Mg efflux. Indeed, the Mg level was decreased in interstitial fluid from scWAT of adipocyte‐specific knockout CNNM4 (Cnnm4^fl/fl^×Adipoq‐Cre, CNNM4^Adipoq‐cKO^) comparing to WT mice under cold exposure (Figure 3J). On the contrary, CNNM4 overexpression in scWAT by Adv‐CNNM4 local injection increased interstitial Mg secretion under cold exposure (Figure 3K). These results suggested that CNNM4 mediated ADRB3‐stimulated Mg release from thermogenic adipocytes.

### 2.4 CNNM4 Promotes M2 Macrophage Polarization and Thermogenesis

**Figure 4:**
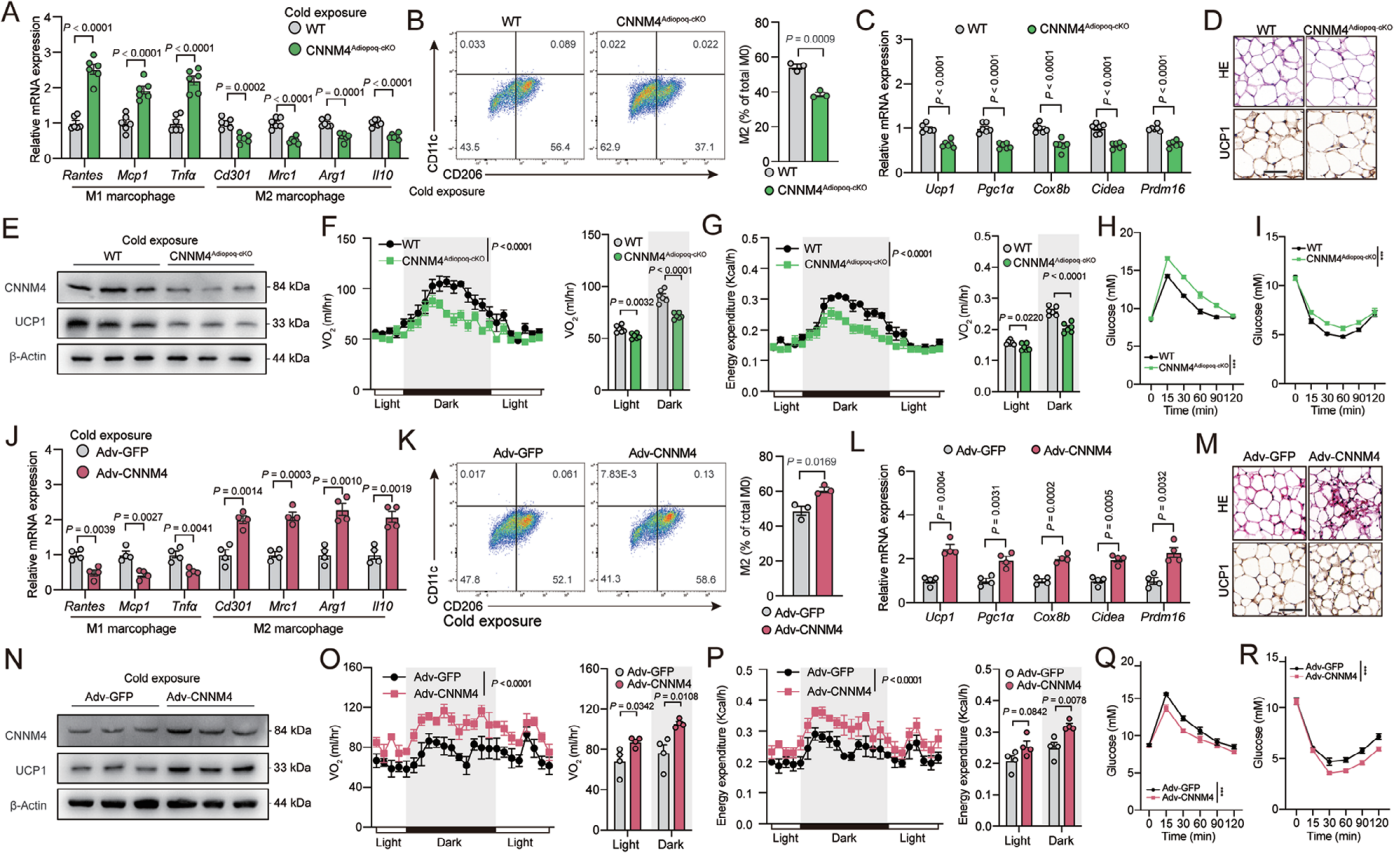
CNNM4 promotes M2 macrophage polarization and thermogenesis. A) Representative M1 and M2 macrophage marker gene expression (*n* = 6) and B) flow cytometric plots and quantification demonstrate the numbers of M2 macrophages (CD206^+^/CD11c^−^) (*n* = 3) in scWAT from CNNM4^Adipoq‐cKO^ or WT mice under cold exposure; C) representative thermogenic genes expression in scWAT from CNNM4^Adipoq‐cKO^ or WT mice (*n* = 6); D) representative H&E staining (Top) and UCP1 immunohistochemistry (Down), E) UCP1 and CNNM4 protein expression in scWAT, and F) VO_2_, G) energy expenditure of CNNM4^Adipoq‐cKO^ or WT mice (*n* = 6), Scale bar, 50 µm; H) glucose tolerance and I) insulin tolerance tests of CNNM4^Adipoq‐cKO^ or WT mice under normal diet condition (*n* = 6); significance symbols presented in GTT and ITT panels relate to AUC analysis. J) Representative M1 and M2 macrophage marker gene expression (*n* = 4) and K) flow cytometric plots and quantification demonstrate the numbers of M2 macrophages (CD206^+^/CD11c^−^) (*n* = 3) in scWAT from Adv‐GFP or Adv‐CNNM4‐local‐injected mice under cold exposure; L) representative thermogenic genes expression in scWAT from Adv‐GFP or Adv‐CNNM4‐local‐injected mice under cold exposure (*n* = 4); M) representative H&E staining (Top) and UCP1 immunohistochemistry (Down), N) UCP1 and CNNM4 protein expression in scWAT, and O) VO_2_, P) energy expenditure of Adv‐GFP or Adv‐CNNM4‐local‐injected mice under cold exposure (*n* = 4), Scale bar, 50 µm; Q) glucose tolerance and R) insulin tolerance tests of Adv‐GFP or Adv‐CNNM4‐local‐injected mice under normal diet condition (*n* = 4). Data were expressed as means ± SEM. (A)–(D), (F)–(I), (J)–(M), (O)–(R) were calculated by unpaired two‐tailed Student's *t*‐test, or (F), (G), (O), and (P) were calculated ANCOVA with body weight as covariant.

We next tried to assess the function of CNNM4 on M2 macrophage polarization and thermogenesis in vivo by using CNNM4^Adipoq‐cKO^ mice. We generated primary beige adipocytes from WT and CNNM4^Adipoq‐cKO^ mice and treated them with CL316243. When applied to BMDM, M2 macrophage polarization was decreased by medium from CL316243‐treated CNNM4^Adipoq‐cKO^ beige adipocytes compared to medium from CL316243‐treated WT beige adipocytes (Figure S10A,B, Supporting Information). Meanwhile, M2 macrophage polarization as detected by marker genes expression and flow cytometry analysis was also decreased in scWAT and BAT from CNNM4^Adipoq‐cKO^ compared to WT littermates under cold exposure (**Figures** **4**A and S10C,D, Supporting Information).

M2 macrophages have been reported to promote thermogenesis and improve systemic insulin sensitivity, although the exact mechanism is still under debate.^[^ ^41^, ^42^ ^]^ Indeed, increased adiposity and decreased UCP1 protein levels, thermogenic gene expression were observed in scWAT from CNNM4^Adipoq‐cKO^ mice compared to WT littermates under cold exposure (Figure 4C–E, and Figure S11, Supporting Information). As a result, whole body indirect calorimetry analysis revealed lower oxygen consumption (VO_2_), and energy expenditure in CNNM4^Adipoq‐cKO^ mice than control littermate under cold exposure (Figure 4F,G). Food intake was unaltered in WT and CNNM4^Adipoq‐cKO^ mice (Figure S12, Supporting Information). Furthermore, insulin sensitivity/glucose homeostasis and insulin signaling in scWAT, liver, and muscle as shown by the phosphorylation of AKT and GSK‐3β were also impaired in CNNM4^Adipoq‐cKO^ mice (Figure 4H,I and Figures S13 and S14, Supporting Information). Similar results were also obtained in CNNM4^Fabp4‐cKO^ (Cnnm4^fl/fl^×Fabp4‐creERT) compared to WT littermates under cold exposure (Figure S15, Supporting Information).

On the contrary, we injected Adv‐GFP or Adv‐CNNM4 locally in the scWAT, which led to nearly sixfold overexpression of CNNM4 in scWAT but not in liver, muscle, BAT, or brain (Figure S16A, Supporting Information). We verified that Adv‐CNNM4 infected adipocytes (Perilipin positive) but not sympathetic neurons (TH positive), vessels (CD31 positive), and immune cells (CD45 positive) (Figure S16B, Supporting Information). CNNM4 overexpression in scWAT resulted in increased M2 macrophage polarization, UCP1 protein levels, thermogenic gene expression, VO_2_, energy expenditure under cold exposure, and improved insulin sensitivity/glucose homeostasis (Figure 4J–R and Figure S17, Supporting Information). Moreover, there were no detectable differences of ADRB3 expression levels and ADRB3 signalings in scWAT from both CNNM4^Adipoq‐cKO^ and Adv‐CNNM4‐injected mice, thus precluding the autonomous effect of CNNM4 on adipocytes (Figure S18, Supporting Information). These observations demonstrated that CNNM4 promoted M2 macrophage polarization, thermogenesis and insulin sensitivity in vivo.

As is shown in Figure S18 (Supporting Information), basal respiration, maximal respiration, and proton leak oxygen consumption rate (OCR) were significantly decreased in mature adipocytes from CNNM4Adipoq‐cko mice compared to that from WT mice, while the mitochondrial respiration was promoted in mature adipocytes from CNNM4‐overexpressed mice than the control counterpart (Figure S19A,B, Supporting Information). Moreover, to complement the predominantly gene expression‐based characterization of macrophages, we also measured the macrophage metabolic profiles using Seahorse. Consistent with our previous study, higher basal respiration, maximal respiration, and proton leak OCR can be observed in ATMs from CNNM4‐overexpressed mice or AMI mice, while inhibited mitochondrial respiration was observed in ATM from CNNM4Adipoq‐cko mice (Figure S19C,D, Supporting Information).

### 2.5 CNNM4 Expression Is Associated with Obesity

**Figure 5:**
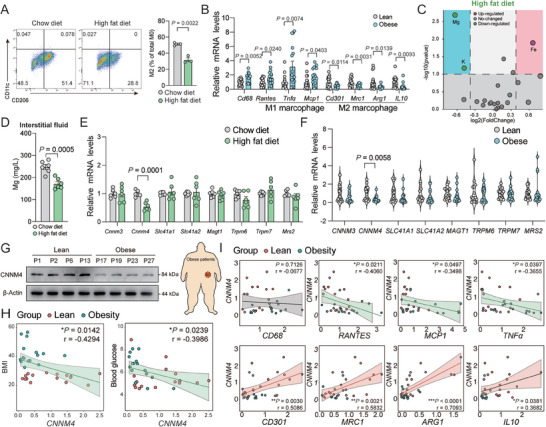
CNNM4 expression is associated with obesity. A) Representative flow cytometric plots and quantification demonstrate the numbers of M2 macrophages (CD206^+^/CD11c^−^) in scWAT of mice fed with HFD or regular diet (RD) for 12 weeks (*n* = 3); B) representative M1 and M2 macrophage marker gene expression in scWAT from lean and obese human individuals (16 vs 16); C) volcano plot of ions altered in scWAT‐derived interstitial fluid from HFD or RD‐fed mice (*n* = 4); D) Mg levels in scWAT‐derived interstitial fluid from HFD or RD‐fed mice (*n* = 6); E) representative Mg transporters (*Mrs2, Cnnm4, Cnnm3, Slc41a1, Slc41a2, Magt1, Trpm6*, and *Trpm7*) gene expression in scWAT from HFD or RD‐fed mice (*n* = 6) or F) lean and obese human individuals (16 vs 16); G) representative CNNM4 and UCP1 protein expression in scWAT from lean and obese human individuals; H) the correlation between BMI and blood glucose, I) representative M1 and M2 macrophage marker gene expression (*CD68, RANTES, MCP1, TNFα, CD301, MRC1, ARG1*, and *IL10*) with CNNM4 expression in lean and obese human individuals (*n* = 32). Data were expressed as means ± SEM. (A), (B), and (D)–(F) were calculated by unpaired two‐tailed Student's *t*‐test or (H) and (I) were analyzed by Pearson correlation analysis.

Obesity is featured with low M2 macrophage polarization in adipose tissue (**Figures** **5**A,B and S20A–C, Supporting Information) and low energy expenditure. Whether CNNM4‐mediated M2 macrophage polarization plays a role in the development of obesity is an interesting topic that requires to be clarified. Indeed, we noted that Mg levels were decreased in the interstitial fluid of scWAT but not in the plasma from HFD‐fed mice (Figure 5C,D and Figure S20D, Supporting Information). CNNM4 mRNA levels were also decreased in scWAT from HFD‐fed mice (Figure 5E). We also confirmed the decreased CNNM4 expression in human scWAT samples from obese individuals and a negative correlation of CNNM4 mRNA levels in scWAT with BMI and blood glucose levels was observed (Figure 5F–H). Importantly, we noticed that CNNM4 expression was positively correlated with M2 macrophage marker genes such as CD301, MRC1, ARG1, and IL10, while negatively correlated with M1 macrophage marker genes such as RANTES, MCP1, and TNFα, indicating that decreased CNNM4 expression levels and Mg secretion might contribute to the progress of obesity. Moreover, there was no statistic difference at the expression of activated macrophage marker CD68 (Figure 5I).

To explore the potential anti‐obesity effects of CNNM4, We injected a recombinant adenovirus‐associated virus (AAV) expressing CNNM4 in scWAT of HFD‐fed mice, which led to overexpression of CNNM4 in scWAT but not in liver, muscle, BAT, or brain (Figure S21A, Supporting Information). We verified that AAV‐CNNM4 infected adipocytes (Perilipin positive) but not sympathetic neurons (TH positive), vessels (CD31 positive), and immune cells (CD45 positive) (Figure S21B, Supporting Information). CNNM4 overexpression in scWAT ameliorated HFD‐induced body weight gain, adiposity (Figure S18C–E, Supporting Information). Thermogenic genes expression and UCP1 protein levels in scWAT were increased (Figure S21F,G, Supporting Information). Although food intake was unaltered, VO_2_ and energy expenditure in AAV‐CNNM4‐infected HFD‐fed mice was promoted (Figure S21H–J, Supporting Information). Moreover, overexpression of CNNM4 also improved insulin sensitivity/glucose homeostasis (Figure S21K,L, Supporting Information). On the contrary, CNNM4^Adipoq‐cKO^ mice presented an increased body weight gain with similar food intake compared to WT mice under HFD feeding (Figure S22, Supporting Information).

We then investigated whether local administration of Mg in scWAT could serve as a strategy against obesity. Indeed, local injection of MgCl_2_ solution into scWAT of HFD‐fed mice for 7 d promoted M2 macrophage polarization in scWAT (Figure S23A,B, Supporting Information). Meanwhile, UCP1 protein levels and thermogenic gene expression were also increased (Figure S23C,D, Supporting Information). VO_2_ and energy expenditure as well as insulin sensitivity/glucose homeostasis and insulin signaling in scWAT, liver and muscle were improved in MgCl_2_‐injected mice versus PBS‐injected mice (Figure S23E–K, Supporting Information). Of note, Mg administration also decreased HFD‐induced body weight gain without any influence on food intake (Figure S23L,M, Supporting Information). These results clearly showed that CNNM4 expression or Mg supplement could serve as a new strategy against obesity.

### 2.6 AMI Ameliorates Obesity

Acupuncture is a Chinese approach against various diseases, especially weight control.^[^ ^43^, ^44^, ^45^ ^]^ Based on multicenter data, long‐term acupoint stimulation, namely, embedding acupuncture, by implanting compatible and self‐degradable materials can improve triglyceride levels and subcutaneous adipose tissue without adverse events.^[^ ^46^ ^]^ As one of the modified acupunctural techniques, the implantation of absorbable materials presented better therapeutic effects and notably decreased treatment frequency from approximately twice a week to twice a month.^[^ ^47^ ^]^ The ability to conserve time and costs makes it an attractive strategy to manage obesity. We further optimized the Mg delivery system by inviting an adipose magnesium wire implantation (AMI) system for long‐term consistently release of Mg in scWAT with adipose titanium wire implantation (ATI) which would not release any ions as a control. We first characterized the function of AMI on M2 macrophage polarization in vitro by applying extracts of Mg wire onto BMDMs and THP1 cells. An increase of M2 macrophage marker genes expression and a decrease of M1 macrophage marker genes expression were observed in both BMDMs and THP1 cells (Figure S24, Supporting Information).

Next, we implanted AMI in bilateral sides of the scWAT in HFD‐fed mice. The typical degradation mode of Mg wire implants was through a corrosion process in the physiological environment, which proceeds by an electrochemical reaction with electrolyte to produce Mg^2+^ continually (Figure S25A,B, Supporting Information). The highest daily release of Mg^2+^ happened on the first day of implantation (about 0.4 mg), and the average daily release within 28 d of implantation was about 0.086 mg (Figure S25B, Supporting Information). The surface of Mg wire implants was covered with degradation products comprised of Mg, C, O, Ca, and P elements providing a good biocompatibility (Figure S25C,D, Supporting Information). To explore the potential toxicity of AMI on other tissues in the body, we detected the histological images of internal organs, including intestine, lung, pancreas, heart, liver, kidney, and spleen. No inflammatory cell infiltration, histopathological changes (degeneration and necrosis), or corrosion product accumulation were observed, indicating normal histological morphologies in these tissues (Figure S26A, Supporting Information). In addition, the biochemical analysis of peripheral blood showed no significant differences between AMI group and the ATI group (Figure S26B, Supporting Information). Importantly, the concentration of serum Mg was not influenced by AMI, suggesting an adipose tissue local function of Mg wire implants (Figure S26B, Supporting Information). These results indicated the biocompatible and biosafety of the AMI approaches.

**Figure 6:**
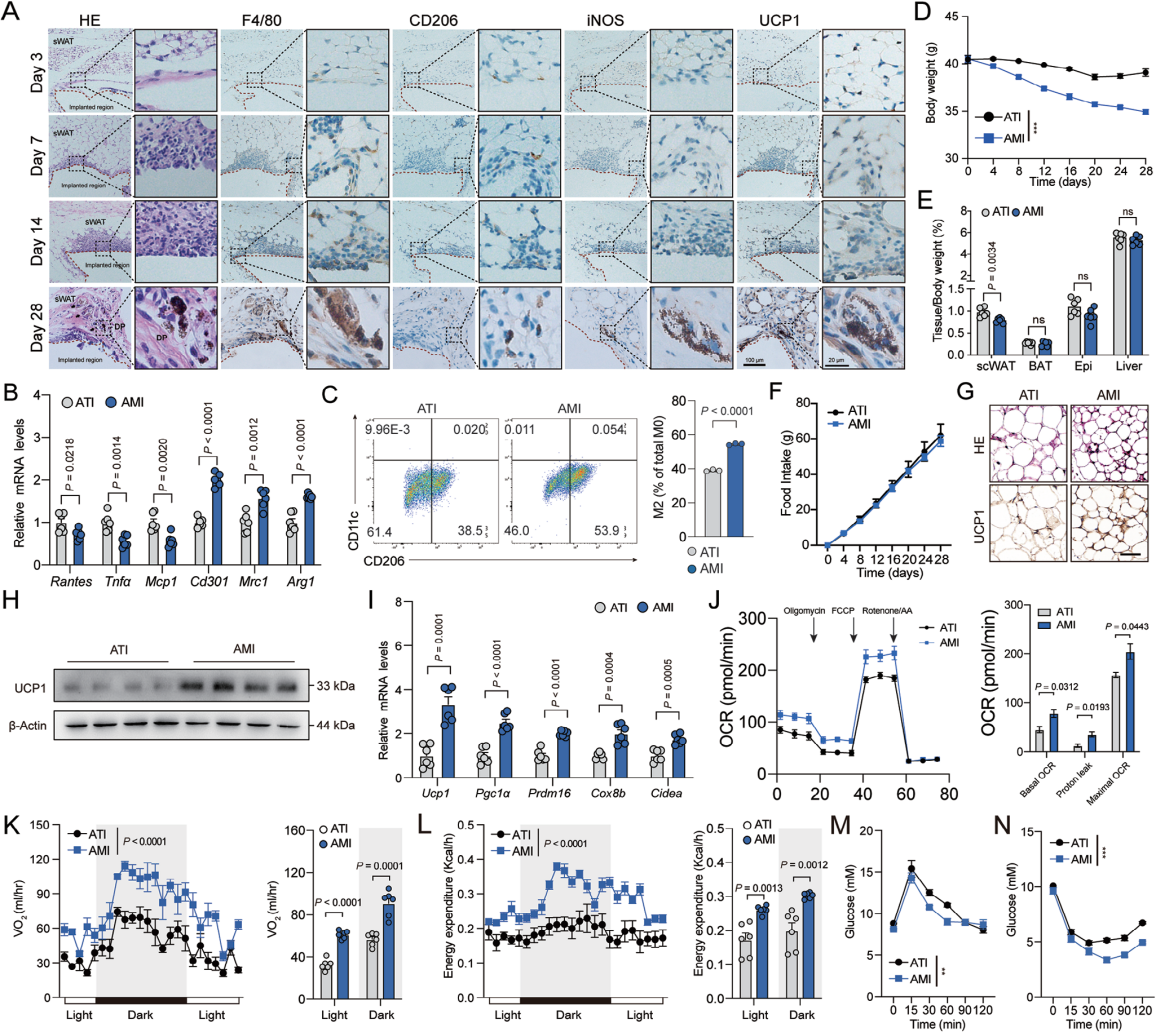
AMI ameliorates obesity. A) Representative H&E staining and immunohistochemical staining (F4/80, CD206,iNOS, and UCP1) of scWAT at indicated time points after Mg wire implantation (AMI). Scale bar, 100 or 20 µm; B) representative M1 and M2 macrophage marker gene expression (*n* = 6) and C) flow cytometric plots and quantification demonstrate the numbers of M2 macrophages (CD206^+^/CD11c^−^) (*n* = 3) in scWAT of HFD‐fed mice after four‐week ATI or AMI; D) body weight, E) indicated tissue weight, and F) food intake in HFD‐fed mice after four‐week ATI or AMI (*n* = 6); G) representative H&E staining (Top) and UCP1 immunohistochemical (Down) staining, H) UCP1 protein expression, I) representative thermogenic genes expression, and J) OCR of mature adipocytes from HFD‐fed mice after four‐week ATI or AMI (*n* = 6). Scale bar, 50 µm; K) VO_2_ and L) energy expenditure of HFD‐fed mice after four‐week ATI or AMI (*n* = 6); M) glucose tolerance and N) insulin tolerance tests of HFD‐fed mice after four‐week ATI or AMI (*n* = 6); significance symbols presented in GTT and ITT panels relate to AUC analysis. Data were expressed as means ± SEM. (B), (C), (E), (G), and (I)–(N) were calculated by unpaired two‐tailed Student's *t*‐test; (D), (F), and (J) were calculated by two‐way ANOVA with Bonferroni's multiple comparison test; (K) and (L) were analyzed by ANCOVA with body weight as covariant.

M2 macrophage polarization was increased not only in the area of AMI but in the whole tissue, suggesting that AMI released Mg could spread to the whole adipose tissue (**Figures** **6**A–C and S27A, Supporting Information). Furthermore, we also detected the M2 macrophage polarization marker genes after AMI with normal chow diet. Consistent with the results in HFD‐fed mice, AMI‐released Mg could also promote M2 macrophage polarization in this setting (Figure S27, Supporting Information). Meanwhile, AMI ameliorated obesity and adiposity in scWAT without any influence on food intake (Figure 6D–G). UCP1 protein levels as detected by both immunoblot and immunostaining and thermogenic genes expression were also upregulated in scWAT (Figure 6G–I). We also explored the distribution of UCP1 expression with whole tissue scan and observed a broad positive signal throughout scWAT in consistent with the M2 macrophage polarization data (Figure S27B, Supporting Information). We further used tissue ex vivo respiration measurement with seahorse to directly test the OCR in mature adipocytes from scWAT. As is shown in Figure 6J, higher basal respiration, maximal respiration, and proton leak OCR can be observed in mature adipocytes from AMI mice than ATI mice, demonstrating an increased thermogenesis after magnesium implantation strategy. Meanwhile, VO_2_ and energy expenditure were increased in AMI mice compared to ATI mice (Figure 6K,L). Insulin sensitivity/glucose homeostasis and insulin signaling in scWAT, liver, and muscle were also improved in AMI mice (Figure 6M,N, and Figure S29, Supporting Information). Importantly, the beneficial effect of AMI relied on macrophages and mTORC2 signaling as macrophage depletion by Clodronate liposome or inhibition of macrophage mTORC2 activation with adeno‐associated virus (AAV)‐CD68‐shRictor injection in scWAT abolished these effects (Figure S30, Supporting Information).

Taken together, we have identified Mg as a thermogenic adipocyte‐secreted factor to promote M2 macrophage polarization and thermogenesis in adipose tissue. ADRB3‐PKA‐CREB cascade stimulates CNNM4 expression which mediates the efflux of Mg in thermogenic adipocytes. Mg in turn binds to the DFG motif in mTOR and facilitates the activation of mTORC2 complex which promotes M2 macrophage polarization. Importantly, overexpression of CNNM4 or supplementation of Mg in scWAT promotes thermogenesis and ameliorates obesity. The AMI approach facilitates the Mg delivery in scWAT and could serve as a new approach against obesity.

## 3 Discussion

**Figure 7:**
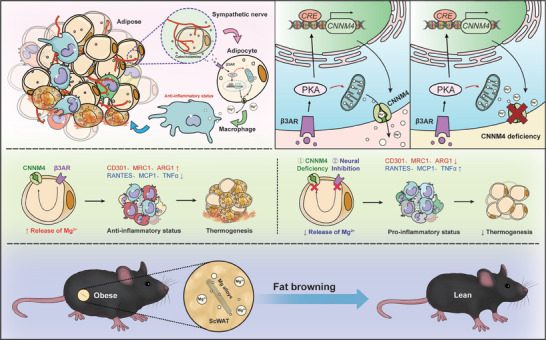
Schematic overview of main findings.

Here, we identified a new mechanism for the reciprocal regulation of thermogenic adipocytes and macrophages to boost thermogenesis. Under cold exposure, sympathetic innervation promoted adipose thermogenesis through ADRB3 which activated PKA signaling to increase CNNM4 expression through CREB. CNNM4‐mediated Mg efflux from thermogenic adipocytes further activated mTORC2 signaling and promoted M2 macrophage polarization. Under obese conditions, lower CNNM4 expression accounted for the decreased Mg secretion which impaired M2 macrophage polarization‐induced thermogenesis and deteriorated the progress of obesity. Thus, means of promoting this feedback loop by either CNNM4 expression, Mg supplement, or AMI could serve as valuable approaches to combat obesity (**Figure** **7**).

Immune cells, especially macrophages, in adipose tissue play critical roles in adipose thermogenesis. Macrophages are the most abundant cell types in the SVF of adipose tissues, accounting for ≈10% and 40% in lean and obese states, respectively.^[^ ^48^ ^]^ While proinflammatory cytokines increase M1 macrophage polarization, which suppresses the induction of UCP1 expression and thermogenesis in BAT and WAT,^[^ ^49^, ^50^, ^51^, ^52^ ^]^ type 2 cytokines trigger M2 macrophage polarization, which promotes adipose thermogenesis through unclear mechanisms.^[^ ^53^ ^]^ Numerous chemokines and secreted factors could directly or indirectly promote polarization and activation of M2 macrophage and subsequent thermogenesis.^[^ ^16^, ^17^, ^18^, ^19^ ^]^ For example, eosinophils recruited by type 2 innate lymphoid cells (ILC2s) secrete interleukin‐5 (IL‐5) and IL‐13 to activate M2 macrophages, which leads to thermogenic activation of beige adipocytes.^[^ ^54^, ^55^, ^56^ ^]^ Meteorin‐like (Metrnl), a circulating factor that is induced in muscle by exercise stimulates thermogenesis through an eosinophil‐dependent M2 macrophage activation.^[^ ^19^ ^]^ These reports all show that means to promote M2 macrophage polarization serve as important strategies to promote thermogenesis; however, the mechanism by which M2 macrophages promote thermogenesis has been less well studied. One study reported that adipose tissue macrophages (ATMs) released NE promotes thermogenesis,^[^ ^41^ ^]^ while another report demonstrated that M2 macrophages lack tyrosin hydroxylase (TH) to make catecholamines and deletion of tyrosine hydroxylase expression or inhibition of NE synthesis in M2 macrophages fails to impair thermogenesis of adipose tissue.^[^ ^57^ ^]^ Meanwhile, a new identified sympathetic neuron‐associated Cx3cr1^+^ macrophages decrease thermogenesis by uptake and clearance of catecholamines. Although there is a debate on the macrophage‐derived NE, the association between M2 macrophage and adipose thermogenesis has been revealed in accumulative evidence.^[^ ^19^, ^54^, ^56^ ^]^ In addition, a recent study reported that M2 macrophages intensify sympathetic nerve function by secreting Slit3 to promote adipose thermogenesis, which serves as a mediator for the communication among macrophages, sympathetic nerves, and adipose thermogenesis.^[^ ^58^ ^]^ The exact mechanism of how M2 macrophages promote thermogenesis will need further investigation.

Dynamic crosstalk between adipocytes and macrophages is critical for adipose thermogenesis. Amlexanox promotes the expression of IL‐6 in adipocytes through cAMP signaling. IL‐6‐dependent Tyr705 phosphorylation of STAT3 (pY705 STAT3) in adipose tissue macrophages results in upregulation of Il4ra and sensitization to IL‐4 signaling. As a result, M2 macrophage polarization is increased.^[^ ^59^ ^]^ Thermogenic adipocytes‐enriched MiR‐182‐5p promotes adipose thermogenesis via an M2 macrophage‐dependent mechanism involving FGF21^[^ ^60^ ^]^. Recent findings also reported that mitochondria could transfer from adipocytes to macrophages to promote M2 macrophage polarization and adipose thermogenesis.^[^ ^61^ ^]^ In this study, we identified Mg as a new mediator for the crosstalk between adipocytes and macrophages. Although Qiao et al. have reported that local Mg levels play a central role in immunomodulation in macrophages,^[^ ^32^ ^]^ we further demonstrated that ADRB3‐PKA‐CREB cascade promoted the release of Mg from thermogenic adipocytes through CNNM4 and Mg stimulated M2 macrophage polarization via mTORC2. Importantly, this mechanism promoted adipose thermogenesis and impaired Mg secretion by CNNM4 downregulation contributed to the progress of obesity. Thus, not only protein factors, miRNA, and mitochondria but also ions could link adipocytes and macrophages together and thorough investigations of the crosstalk between adipocytes and macrophages would provide us with new strategies to combat against the world‐wild prevalence of obesity. Moreover, the diversity of ATMs should also be considered. The perivascular macrophages (PVMs) (CD206^+^/CD163^+^/LYVE1^+^/TIM4^+^) has been observed in scRNA‐seq studies and dominate as a proportion of all ATMs. PVM initially originates from macrophages derived from the yolk sac or erythromyeloid progenitors, but are eventually replaced over time by monocyte‐derived macrophages.^[^ ^62^ ^]^ A second ATM type of non‐PVM ATMs has also been identified; this subtype has a LYVE1^low^MHC‐II^hi^ phenotype that is enriched for pathways related to inflammation and chemotaxis. This population of cells includes sympathetic neuron‐associated macrophages, which mediate the clearance of noradrenaline and antagonize brown and beige adipocyte activity.^[^ ^63^ ^]^ Moreover, the capacity of immune cells to respond to microenvironmental nutrient stress is evident in the identification of lipid‐associated macrophages (LAMs). LAMs represent a type of macrophage programming that is seen in multiple tissue and disease contexts where fatty acid availability is in excess or is inappropriately regulated.^[^ ^64^ ^]^ Additional components of this “immune‐centric” branch of immunometabolism might include how nutrients and diet reshape the intestinal microbiome and gut immunity and contribute to dysbiosis.^[^ ^65^ ^]^ However, LAMs are largely absent from lean mice and accumulate in visceral depots, such as peri‐gonadal adipose tissue, in obese mice.^[^ ^64^ ^]^ Due to the majority of macrophage dominated within adipose tissues derived from bone marrow, we mainly explore the role of Mg on the BMDMs in this study. Tissue resident macrophages might also be regulated by Mg and the contribution of tissue resident macrophages on different aspects of the thermogenic regulation by Mg is an interesting topic and will need further investigation.

Mg deficiency is a risk factor for obesity and type2 diabetes,^[^ ^66^, ^67^ ^]^ and Mg supplementation could promote systemic insulin sensitivity.^[^ ^68^ ^]^ Mg supplementation is well‐tolerated, but it could cause several side effects. An overdose of intravenous Mg supplement may cause hypotension, drowsiness, muscle weakness, respiratory depression, cardiac arrhythmia, coma, and even death. Oral intake of Mg may influence the absorption of aminoglycosides, bisphosphonates, and tetracylines.^[^ ^69^ ^]^ Therefore, a tissue‐specific method for Mg supplementation may benefit metabolism homeostasis without these side effects. While serum Mg is just a tiny percentage of the total body Mg content, large amount of Mg is stored intracellularly to regulate numerous cellular functions and enzymes, including metabolism and cell signal pathways. In the meantime, intracellular Mg also serves as a reservoir for maintaining physiological extracellular magnesium level.^[^ ^70^ ^]^ Previous study has reported that acutely Mg‐deficiency in rats with Mg‐deficient diet does not affect brown fat thermogenic UCP1 expression.^[^ ^71^ ^]^ However, they did not provide direct evidence that Mg‐deficient diet could decrease the Mg levels in adipose tissue as intracellular pool of Mg might be motivated and secreted to maintain constant levels of Mg in the tissue environment. In our present study, we locally injected Mg^2+^ or implanted degradable magnesium wire within adipose tissues to accurately elevate the Mg levels in the tissue and noted a promotion of UCP1 expression. The inconsistency between these two studies could be explained by the local change of Mg levels in adipose tissue under different experimental conditions.

In this study, we achieved localized AMI in scWAT by implanting biodegradable Mg based on the research that Mg, as novel implanted devices, has been clinically used in orthopedics.^[^ ^33^, ^72^, ^73^ ^]^ With great biosafety, biocompatibility and biodegradability in vivo, AMI did not show any broad influences on the physiology status of the mice: serum Mg concentration was not influenced; potential toxicity of AMI on internal organs, including intestine, lung, pancreas, heart, liver, kidney, and spleen was not observed; biochemical analysis of peripheral blood and food intake also showed no significant differences between AMI and ATI group. At the meantime, AMI supported the local, long‐term, and stable release of Mg in adipose tissue, which promoted M2 macrophage polarization and thermogenesis. As a result, AMI presented a great value as a feasible way against obesity and insulin resistance. Whether AMI could be used in human and exert anti‐obesity effects is an interesting topic that requires further investigation.

## 4 Experimental Section

### Human Subjects

Two cohorts including 16 lean persons and 16 obese patients were investigated (clinical characteristics in Table S1, Supporting Information). Subcutaneous fat samples were collected from the Department of Endocrinology and Metabolism Center, Shanghai Tenth People's Hospital (Permit Number: 2017KY209), and used for real‐time reverse quantitative PCR and immunoblot analysis. All studies were approved by the regional board of ethics and informed written consent was obtained from each subject.

### Animals

C57BL/6 mice were purchased from Slack Laboratory Animal Co., Ltd. (SLAC, Shanghai, China). CNNM4^fl/fl^, Adipoq‐Cre, and Fabp4‐creERT mice were purchased from Cyagen Biosciences. DTR‐STOP^fl/fl^ mice were kindly provided by Dr. Siguang Li from School of Medicine, Tongji University. All mice were on the C57BL/6 background. Male mice aged 8–16 weeks were housed under specific pathogen‐free conditions and free access to food and water. All animal experiments were performed according to protocols established by Animal Experiment Committee of Tongji University (Permit No. 2024‐DW‐SB‐223), and in accordance with the guidelines of School of Medicine, Tongji University.

### Generation of Conditional CNNM4 Knockout Mice

CNNM4^fl/fl^ mice were generated on a C57BL/6 background. To obtain conditional CNNM4 knockout mice, homozygous CNNM4^fl/fl^ mice were crossed with hemizygous Adipoq‐Cre mice. Adipoq‐Cre; CNNM4^fl/fl^ mice were obtained after at least two generations of crossing and transformed into conditional CNNM4 knockout (CNNM4^Adipoq‐cKO^) animals. Homozygous Fabp4‐creERT mice, in which the Cre‐mediated recombination is controlled by tamoxifen and is ubiquitous in all tissues. Fabp4‐creERT; CNNM4^fl/fl^ mice were obtained after at least two generations of crossing and transformed into conditional CNNM4 knockout (CNNM4^Fabp4‐cKO^) animals following tamoxifen injections (75 mg kg^−1^, i.p., once a day for 5 d). Mice with the genotype of Adipoq‐Cre; CNNM4^wt/wt^ or Fabp4‐creERT; CNNM4^wt/wt^ were used as controls (CNNM4 WT) and received the same tamoxifen treatment regimen in all studies. Induced knockout of CNNM4 was confirmed by Western blotting.

### Diphtheria Toxin‐Mediated UCP1‐Positive Cell Depletion

UCP1‐Cre; DTR‐STOP^fl/fl^ mice (UCP1‐DTR mice) were injected with Diphtheria toxin or PBS as control (200 ng per 20 g of body weight) once daily for seven consecutive days before sacrifice.

### Cells

SVFs obtained from scWAT of 8‐week‐old male mice were cultured in Dulbecco's Modified Eagle Medium/Nutrient Mixture F‐12 (DMEM/F12) containing 10% fetal bovine serum (FBS) and 1% penicillin‐streptomycin solution. Primary adipocytes were induced by beige cocktail containing 0.5 × 10^−3^ m isobutylmethylxanthine, 125 × 10^−9^ m indomethacin, 2 µg mL^−1^ dexamethasone, 850 × 10^−9^ m insulin, 1 × 10^−9^ m T3, and 0.5 × 10^−6^ m rosiglitazone for 2 d followed by adding medium containing 10% FBS, 850 × 10^−9^ m insulin, 1 × 10^−9^ m T3, and 0.5 × 10^−6^ m rosiglitazone for another 4 d or white cocktail containing insulin (860 × 10^−9^ m), dexamethasone (2 µg mL^−1^), and IBMX (0.5 × 10^−3^ m) for 2 d followed by medium with insulin (860 × 10^−9^ m) for another 4 d. To investigate the phospho‐CREB and CNNM4 levels, adipocytes were pretreated with L748337 or H89 for 30 min and followed by CL316243 treatment for another 30 min.

BMDMs isolated from the femur and tibia of C57BL/6 mice were maintained in RPMI 1640 medium with 10% FBS, 1% penicillin‐streptomycin, and 25 ng mL^−1^ recombinant murine MCSF (416‐ML, R&D systems).

THP1 and HEK293A cells were obtained from the American Type Culture Collection (ATCC) and maintained in DMEM containing 10% FBS and 1% penicillin/streptomycin. No commonly misidentified cell line was used in this study. All cell lines were routinely tested negative for mycoplasma contamination.

### Magnet‐Activated Cell Sorting

ATMs were separated from scWAT primary cells by magnetic‐immunoaffinity isolation using anti‐F4/80 antibodies conjugated to magnetic beads (MACS Cell Separation System; Miltenyi Biotec, Bergisch Gladbach, Germany). After scWAT isolation, F4/80‐positive cells were separated using positive‐selection columns (LD columns; Miltenyi Biotec, Bergisch Gladbach, Germany), according to the manufacturer's instructions. The isolated ATMs were analyzed in the following experiments.

### Chemicals and Antibodies

The following Chemicals were used in this study: CL316243 (C5976, Sigma‐Aldrich), H89 (B1427, Sigma‐Aldrich), TMRM (I34361, Thermo Fisher Scientific), MitoTracker (40741ES50, YEASEN), FCCP (C2920, Sigma), Isobutylmethylxanthine (I7018, Sigma‐Aldrich), Indomethacin (I8280, Sigma‐Aldrich), Dexamethasone (D4902, Sigma‐Aldrich), Insulin (I0305000, Sigma‐Aldrich), T3 (T2877, Sigma‐Aldrich), Rosiglitazone (R2408, Sigma‐Aldrich), Poly‐l‐lysine (P2636, Sigma‐Aldrich), CsA(59865‐13‐3, MCE), Mag‐Fluo‐4 AM (MX4543‐500UG, MKBio), Pluronic F‐127 (MS4301‐1G, MKBio), Nitrendipine (HY‐B0424, MCE), 2‐aminoethoxydiphenyl borate (2APB) (HY‐W009724, MCE), Ruthenium red (HY‐103311, MCE), FTY720 (HY‐12005, MCE), and MgCl_2_ solution (63069, Sigma‐Aldrich).

The following antibodies were used in this study: TH (AB152, Sigma), UCP1 (72298T, Cell Signaling Tech), pSTAT6 (Tyr641) (56554, Cell Signaling Tech), STAT6 (5379, Cell Signaling Tech), pAKT (Ser473) (9271, Cell Signaling Tech), AKT (ab18785, Abcam), anti‐pGSK‐3β (5558, Cell Signaling Tech), anti‐GSK‐3β (12456, Cell Signaling Tech). Rictor antibody (ab70374, Abcam), mTOR antibody (ab32028, Abcam), Raptor (2280, Cell Signaling Tech), CNNM4 (ab191207, Abcam), β‐actin (A3854, Sigma‐Aldrich), CD206 antibody (ab64693, Abcam), CD68 antibody (sc‐20060, Santa Cruz), F4/80 antibody (sc‐377009, Santa Cruz), horseradish peroxidase (HRP)‐conjugated goat anti‐Rabbit IgG antibody (7074, Cell Signaling), and HRP conjugated goat anti‐mouse IgG antibody (7076, Cell Signaling).

### Wire Extracts Preparation

Metal extracts were prepared by using a serum‐free DMEM with an extraction ratio of 0.5 cm^2^ mL^−1^ in a humidified atmosphere with 5% CO_2_ at 37 °C for 72 h. The original 100% extracts were diluted into 50% and 10% concentrations with RPMI1640 or DMEM complete medium before use.

### Detection of Mg Uptake in Macrophages

BMDMs were incubated in Mg^2+^‐free RPMI 1640 supplemented with 4 × 10^−6^ m Mag‐Fluo‐4 and 1 × 10^−6^ m F‐127 for 30 min. After that, cells were washed with Mg^2+^‐free RPMI 1640. Real‐time images were captured using a spinning confocal microscope (Perkin Elmer Instruments, USA) immediately after the addition of 8.0 × 10^−3^ m MgCl_2_ into the Mg^2+^‐free RPMI 1640.

### Cell Stimulation

Magnesium chloride (MgCl_2_, Sigma‐Aldrich) was used to stimulate BMDMs or THP1 cells. Macrophages were differentiated from THP1 cells using serum‐free DMEM supplemented with 10 ng mL^−1^ phorbol 12‐myristate 13‐acetate (PMA, Sigma‐Aldrich). After 48 h, the THP1‐derived macrophages were further cultured in customized Mg‐free DMEM supplemented with different concentrations of Mg^2+^ (i.e., 0.1, 1, and 5 × 10^−6^ m) for another 24 h to allow full differentiation and polarization. BMDMs were also further cultured in customized Mg‐free RPMI 1640 medium supplemented with different concentrations of Mg^2+^ (i.e., 0.1, 1, and 5 × 10^−6^ m) for another 24 h. The BMDMs kept M‐CSF‐depleted for 24 h for M‐CSF stimulation.

### TRPM7 Inhibition and Blockage

For TRPM7 silencing, BMDMs were transfected with 10 × 10^−9^ m siRNA targeting mice TRPM7 (SR423257, OriGene, USA) following the manufacturer's instructions using siTran1.0 (Ori‐Gene, USA) as the agent. Cells transfected with nonspecific control siRNA (SR30004, OriGene, USA) were used as the control. siRNA transfection efficiency was verified 72 h after the transfection by immunoblotting.

### Flow Cytometry Analysis

scWAT or BAT were removed, rinsed in PBS containing 10 × 10^−3^ m CaCl_2_, digested at 37 °C for 60–90 min in DMEM containing 10 × 10^−3^ m CaCl_2_, 1 mg mL^−1^ collagenase type I (Sigma‐Aldrich, SCR103), filtered through a 70‐µm strainer and centrifuged at 700 *g* for 5 min. SVF pellets were collected, and resuspended in red‐blood‐cell lysis buffer (eBioscience RBC Lysis Buffer) before further analysis. To isolate macrophages, SVF pellets were washed twice with PBS and once with FACS staining buffer and stained with F4/80 APC (Biolegend, 123115, 1:200), CD11b PE (Biolegend, 101207, 1:200), CD11c Percp (Biolegend, 117325, 1:100), CD206 FITC (Biolegend, 141703, 1:200) on ice for 1 h in dark. After staining, cells were washed twice with staining buffer, suspended in flow cytometry buffer (PBS with 0.5% BSA and 2 × 10^−3^ m EDTA) and subjected to BD LSRFortessa Cell Analyzer (BD Biosciences). The gating strategy for isolated ATM from SVF was according to previously described. Data were analyzed using FlowJo software version X.0.7 (Tree Star, Inc.) The gating strategy for isolated macrophage from SVF or BMDMs is shown in Figure S28 (Supporting Information).

### Immunoblotting

Cells and tissues were lysed in RIPA lysis buffer (Thermo Fisher, 89900) containing PMSF (Thermo Fisher, 36978) and protease inhibitors (Thermo Fisher, A32963). Total protein lysates were boiled with loading sample buffer containing 10% SDS‐PAGE. Subsequently, separated proteins were transferred onto PVDF membranes (Amersham International, GE Healthcare). PVDF membrane blots were incubated with blocking solution (5% milk powder in tris‐buffered saline‐tween 20 (TBST) for 1 h, then with primary antibody (in blocking solution) overnight at 4 °C. After several washes in TBST, membranes were incubated with HRP‐conjugated secondary antibodies for 1 h at RT in blocking solution. Membranes were incubated with ECL western‐blotting substrate (Amersham International, GE Healthcare) and imaged by a Chemidoc XRS system or ChemiDOC (Bio‐Rad Laboratories).

### RNA Extraction, Quantitative PCR

Total RNA was extracted from cultured cells or tissues with RNAsimple Total RNA Kit (Tiangen, Shanghai, China), and 1 µg total RNA was reverse transcribed to cDNA using the reverse transcripted using FastQuant RT kit (Tiangen, Shanghai, China) according to the manufacturers’ instructions. Quantitative real‐time PCR was performed using the SuperReal SYBR Green kit (Tiangen, Shanghai, China) on the Lightcycler 96 (Roche, Penzberg, Germany). Experiments were repeated three times and gene expression levels were calculated using the delta‐delta Ct method, after normalization to β‐actin expression. The primers were validated, and the sequences are listed in Table S2 (Supporting Information).

### Local Injection in scWAT

For the local injection in adipose tissue, mice were anesthetized and a lateral incision in skin was made to expose scWAT. One side of scWAT was injected with L748337 (ADRB3 antagonist) (5 mg kg^−1^), another side was injected with solvent as control. Mice were sacrificed 24 h later for next experiment.

### Denervation of Adipose Tissue by 6‐OHDA or Surgical Transection

Pharmacological elimination of sympathetic neurons in scWAT and BAT was performed as described previously.^[^ ^28^, ^74^ ^]^ Briefly, mice were anesthetized and a side incision in skin was made to expose scWAT. 15 µL of 6‐hydroxydopamine (15 µg µL^−1^ freshly prepared in saline) containing 1% sodium metabisulfite was injected at each locus of the scWAT or BAT pad, after which the skin incision was sutured. Seven days after the surgery, mice were subjected to following analysis. Surgical transection of sympathetic neurons in scWAT and BAT was performed as described previously.^[^ ^75^ ^]^

### Conditioned Medium Preparation

For CM collection, after primary adipocyte differentiation, the medium was changed to Mg‐free medium (L210KJ; BasalMedia) for 24 h after cells were washed three times with PBS. Then the cells were cultured with 300 µL fresh medium and collected after PBS, CL316243, or CL316243+L748337 treatment for 12 h. The CM were centrifuged at 15000 *g* for 5 min, and filtered through a 0.22‐mm filter. Primary BMDMs were exposed to fresh medium mixed with CM at a ratio of 1:1 (v/v) for 24 h. In the negative group, primary BMDMs were exposed to fresh medium mixed with medium followed with the same process of CM collection without cell culture at a ratio of 1:1 (v/v) for 24 h.

### Interstitial Tissue Fluid Extraction

To reduce the risk of cell compression, interstitial fluid isolation was modified from previous procedures as described previously.^[^ ^76^, ^77^, ^78^ ^]^ Interstitial tissue fluid was extracted from scWAT of mice acclimated to cold exposure or RT for 2 d or high fat diet for eight weeks. In brief, scWAT was placed in a 20 µm nylon mesh (Millipore Sigma, NY2004700), and fixed in a 1.5 mL tube. Subsequently, tissue was centrifuged 10 min at 800 *g* (4 °C). About 5–6 pieces of adipose tissues weighing 1.0 g were transferred intact to the tube, and results from these are later referred to as intact tissues. Typical interstitial fluid samples were 10 µL. Extracted samples were then subjected to following analysis as described.

### Mg Detection by Mass Spectrometry

Ionomic analyses were performed using an inductively coupled plasma‐mass spectrometer (ICP‐MS) system (Agilent Technologies, Tokyo, Japan). Qualitative analysis was performed by measuring specific mass number of the element (mass‐to‐charge ratio, m/z). Quantitative analysis was carried out using the internal standard method, based on the intensity ratio of the mass spectrum signal of the element to be measured and the mass spectrum signal of the internal standard element in direct proportion to the concentration of the element to be measured. The samples were taken out of the refrigerator and homogenized in ice‐water bath after thawing. Dilution of mouse extracellular fluid of scWAT: added 10 µL of nitric acid to tubes. ICP‐MS analysis was performed after homogenization. Cell culture medium sample: 50 µL of cell culture medium was transferred to an EP tube and 950 µL of 0.8% nitric acid was added. ICP‐MS analysis was performed after homogenization.

### Immunoprecipitations and Kinase Assays

Mouse BMDMs were treated with PBS or Mg for 24 h. Then cells were resuspended on ice with intermediated‐efficacy RIPA buffer for 20 min. A total of 4 µg of the anti‐Rictor, anti‐Raptor, or anti‐mTOR antibody was added to the cleared cellular lysates and incubated with rotation for 90 min. In total 25 µL of protein G‐sepharose was then added and the incubation continued for 1 h. Immunoprecipitates captured with protein G‐sepharose were washed four times with ice‐cold lysis buffer and once with the kinase buffer. For kinase reaction, immunoprecipitates were incubated in a final volume of 15 µL for 60 min at 37 °C in the kinase buffer containing 500 ng recombinant inactive PKB and 500 × 10^−6^ m ATP. The reaction was stopped by the addition of 200 µL sample buffer. The samples were analyzed by immunoblotting.

### Bioinformatic Analysis

To compare the changes in Mg transmembrane transporter activity in mouse adipocytes under RT and cold stimulation conditions, single‐cell sequencing data of mouse adipose stromal vascular fraction were obtained from the study of Rajbhandari et al.^[^ ^40^ ^]^ The study was divided into a variety of different experimental conditions, and considering that mice will have cold stimulation adaptation, in this study the 24‐h cold stimulation and RT groups for comparison were only included. Data were obtained from GSE133486^[^ ^40^ ^]^ (https://www.ncbi.nlm.nih.gov/geo/query/acc.cgi?acc = GSE133486), and the Mg transmembrane transporter activity gene set was obtained from the GSEA website (https://www.gsea‐msigdb.org/gsea/msigdb/index.jspGOMF_MAGNESIUM_ION_TRANSMEMBRANE_TRANSPORTER_ACTIVITY). Data were preprocessed according to the methodological section of Rajbhandari et al.,^[^ ^40^ ^]^ and a total of 12 824 high‐quality cells were obtained. Subsequent analysis was done using the R package “Seurat”^[^ ^79^ ^]^ and the gene barcode matrix was normalized using the “LogNormalize” method with the “NormalizeData” function. The first 2000 variable genes were identified using the “vst” method in the “FindVariableFeatures” function. The gene expression matrix was scaled and centered using “ScaleData.” Principal component analysis (PCA) was performed to reduce the dimensionality using the top 25 principal components. A total of 1625 adipocytes were identified based on the marker gene from Rajbhandari et al. The cells were scored for gene sets using the “AddModuleScore” function, and the results were presented using violin plots and statistical tests based on nonparametric tests.

### Chromatin Immunoprecipitation‐Quantitative Polymerase Chain Reaction (ChIP‐qPCR)

Chromatin immunoprecipitation (ChIP) was performed using the Magna ChIP A/G Chromatin Immunoprecipitation Kit (Merck Millipore, Burlington, MA) with an antibody specific for CREB (Abcam, Cambridge, MA) or normal rabbit IgG (Santa Cruz Biotechnology, Santa Cruz, CA). Following ChIP, quantitative PCR was utilized to amplify and quantify the immunoprecipitated DNA using primers for a CREB binding site within CNNM4 promoter region. The ChIP‐qPCR values were normalized to that of input control and represented as fold enrichment relative to the anti‐normal rabbit IgG control.

### Histological Analysis

Dissected adipose tissues were fixed in 10% neutral buffered formalin (Sigma‐Aldrich, HT501128‐4L) overnight at RT and embedded in paraffin. 5‐µm tissue sections were stained with hematoxylin and eosin (Beyotime, C0105S) according to manufacturer's instructions. The images were acquired using the OLYMPUS Microscope. For tissue immunofluorescence, slides were stained with CD206 antibody (1:200) and F4/80 (1:200) overnight at 4 °C and subsequently stained with secondary antibody for 2 h at RT. For cell Immunofluorescence, BMDM or THP1 were washed with PBS for three times and fixed with 4% paraformaldehyde for 15 min at RT, rinsed with PBS and then exposed to 0.1% Triton X‐100 and 5% BSA in PBS for 1 h. Incubation with CD206 antibody (1:200) and CD68 antibody (1:200) diluted in PBS was performed overnight at 4 °C and subsequently stained with secondary antibody for 2 h at RT. Imaging was obtained using the OLYMPUS Microscope.

### Mitochondrial Membrane Potential, Mitochondria and Intracellular Mg Staining

At day 6, after differentiation of beige adipocytes, cells were stained with MitoTracker (300 × 10^−9^ m, 30 min), Mag‐Fluo‐4 AM (5 × 10^−6^ m, 40 min), or TMRM (100 × 10^−6^ m, 30 min). Cells were washed with PBS and subsequently treated with CL316243 (2 mg mL^−1^), H89 (10 × 10^−6^ m), FCCP (5 × 10^−6^ m), or CsA (100 × 10^−9^ m). Cells were then visualized by OLYMPUS Microscope.

### Adenoviral Amplification and Injection

Adv‐CNNM4 and Adv‐GFP were was generated using the AdEasy system.^[^ ^80^ ^]^ Briefly, linearized shuttle vector containing full‐length mouse cDNA for CNNM4 and GFP was transformed into E. coli BJ5183AD cells containing the adenoviral backbone plasmid pAdEasy‐1 for homologous recombination. Positive recombinants were linearized and transfected into HEK293A cells for virus packaging and propagation. Adenoviruses expressing the candidate gene were purified by Fast Trap Adenovirus Purification and Concentration Kit (EMD Millipore), according to the manufacturer's instruction. For the local injection in adipose, 8‐week‐old male mice were anesthetized and a lateral incision in skin was made to expose scWAT. Both sides of scWAT were injected with Adv‐CNNM4 (10^10^ phage‐forming units), Adv‐GFP was used as a control. Seven days later, mice were executed for experiments.

### Macrophage Depletion in Adipose Tissues

Before macrophage depletion, 8‐week‐old mice were implanted with titanium or magnesium wire bilaterally in scWAT pads. Two days later, these mice were subcutaneously injected with 200 µL clodronate liposomes (5 mg mL^−1^) every 4 d for three times until euthanization two weeks later. Control liposomes were paired to the clodronate liposome‐treated counterparts. Afterward, mice were monitored for changes in body weight, fat mass, cold tolerance, and oxygen consumption, then mice were sacrificed and tissues were dissected for further analysis.

### Local Delivery of Adeno‐Associated Virus in Adipose Tissues

AAV vector‐mediated knockdown of mouse macrophages shRNA targeting Rictor (AAV‐CD68‐shRictor) and scrambled control (AAV‐CD68‐scramble) were constructed, amplified, and purified by Hanbio biotechnology (Shanghai, China). A total of 50 µL of 1 × 10^9^ Vg µL^−1^ of each AAV diluted in PBS was injected into the scWAT pads of mice. Mice were monitored for changes in body weight, fat mass, cold tolerance, and oxygen consumption, then mice were sacrificed and tissues were dissected for further analysis.

### Primary Mature Adipocyte Extraction

Subcutaneous white adipose tissue was carefully excised, ensuring to minimize contamination from other tissues like the skin. The excised adipose tissues were finely minced using scissors and then incubated in a solution with collagenase, typically Type I, at 37 °C in a shaking water bath for 60 min. The enzyme breaks down the extracellular matrix, releasing mature adipocytes. After digestion, samples were filtered and centrifuged to separate mature adipocytes from other cell types and debris. Mature adipocytes float due to their lipid content. The mature adipocytes were washed several times with a buffer like PBS to remove collagenase and other contaminants. The suspension of mature adipocytes was introduced in a chamber and stained with methylene blue. Mature adipocytes were identified and counted by an inverted microscope.

### Mitochondrial Stress Analysis

The metabolic profile of mature adipocytes and ATMs was evaluated by the mitostress test and Seahorse technology that allows real‐time evaluation of changes in OCR, measures of oxidative phosphorylation. Real‐time OCR was measured using the XFe96 extracellular flux analyser (Agilent). Briefly, ATMs were seeded at 5 × 10^4^ cells per well in XF96 cell culture microplates and cultured overnight for attachment. Before the assay, cells were incubated in Seahorse assay medium (10 × 10^−3^ m glucose, 2 × 10^−3^ m glutamine, 10 × 10^−3^ m pyruvate). XFe96 cell culture microplate was washed and the final volume of 180 µL of Seahorse assay medium was added to the cell. The means to measure respiration were loaded onto the drug ports of a hydrated sensor cartridge in the following order: oligomycin (1 × 10^−6^ m), FCCP (5 × 10^−6^ m), and antimycin A + rotenone (1 × 10^−6^ m).

Mature adipocytes were centrifuged at room temperature at 200× *g* for 5 min and resuspended in warmed assay medium. Then the cell suspension was inhaled into a hemocytometer. The cells in the counting plate were observed at low power under an optical microscope to obtain the 5 × 10^4^ of cells in 50 µL of assay medium per well. The cells were seeded into the room‐temperature Cell‐Tak‑coated Seahorse XF96 Cell Culture Microplates (for better cell adhesion). The plates were transferred to a 37 °C incubator not supplemented with CO_2_ for 30 min to ensure that the mature adipocytes have completely attached. After visually confirming that most of the mature adipocytes were stably adhered to the culture surface, 130 µL warm assay medium was added along the side of each well. With another 20 min of incubation, the cell plates were examined in a Seahorse XFe96 analyzer.

Different mitochondrial respiratory states were investigated through sequential addition of diverse compounds that modulate or inhibit mitochondrial respiration differently. First, “basal OCR” was measured to indicate cellular respiration under normal physiological conditions. Second, oligomycin (1 × 10^−6^ m) was added to obtain information about mitochondrial “proton leak” and “ATP‐linked OCR.” Following, the uncoupling agent FCCP (5 × 10^−6^ m) was added to acquire the maximum respiratory rate. The difference between maximum capacity and basal OCR indicates the “reserve capacity” of the electron transport chain. Finally, a combination of rotenone (1 × 10^−6^ m) and antimycin‐A (1 × 10^−6^ m) was added to inhibit the activity of complexes III and I respectively, to obtain non‐mitochondrial OCR (NM OCR). Specifically, the OCR parameters are calculated as follows: Basal OCR = OCR before drugs added − NM OCR; Proton leak = OCR after oligomycin − NM OCR; Maximum OCR = OCR after FCCP − NM OCR.

### Metabolic Analysis in Mice

Male mice (eight weeks) were fed with chow diet or HFD (60%, ResearchDiet, D12492) for indicated time. For assessing metabolic parameters, body weight were measured every 4 d. Food intake, locomotor activity, oxygen consumption, and energy expenditure were measured in a subgroup of mice using a Comprehensive Lab Animal Monitoring System (Columbus Instruments, Columbus, OH) and VitalView System (MiniMitter, Bend, Oregon, USA). In brief, mice housed individually with free access to food and water were acclimatized to the metabolic cages for 24 h prior to a 48 h period of automated recordings every 15 min. Sample air from individual cages was passed through sensors to determine O_2_ content by an open‐circuit Oxymax. For cold exposure, mice were housed at 4 °C and core temperature was measured at indicated time. For glucose tolerance test, mice were fasted overnight and injected intraperitoneally with a glucose solution in saline (1.25 g kg^−1^ body weight). For insulin tolerance test, HFD or chow diet mice were fasted for 2 h and received intraperitoneal injection of insulin at 1.25 or 1 U kg^−1^ body weights, respectively. Blood glucose levels were measured at 0, 15, 30, 60, 90, and 120 min after injection with an automated reader (Bayer).

### Adipose Magnesium Wire Implantation

For the AMI system, mice were anesthetized and a lateral incision in skin was made to expose scWAT. The high pure magnesium (HP‐Mg) wires with 99.98% purity were provided by Suzhou Origin Medical Technology Co. Ltd., China. The Mg wire was cut into 5 mm and performed ultraviolet disinfection for 3 h. Bilateral sides of scWAT were implanted with Mg wire, and the titanium wire was considered as nonbiodegradable control. After 2‐d recovery, the implanted mice were used for the following experiments.

### Materials Processing and Degradation Products Characterization

Suzhou Origin Medical Technology Co. Ltd., China, provided the Mg wires with 99.98% purity. After degradation in vivo, the degradation products, residual Mg matrix, and surrounding adipose cells were characterized by scanning electron microscope (SEM, RISE, TESCAN) equipped with energy‐dispersive X‐ray spectroscopy (EDS) for elemental analyses.

### Scanning Electron Microscopy and Elemental Mapping

The samples after implantation with different time were characterized by an SEM (TESCAN Mira3 & AZtec Nordlys Max3). The corrosion morphology image was obtained by using secondary electron (SE) pattern at a working voltage of 5 kV, and the elemental mapping was obtained by using energy‐dispersive spectrometer under the back scattered electron imaging (BSE) pattern at a working voltage of 20 kV.

### Statistical Analysis

All data are shown as mean ± standard error of the mean (SEM) unless otherwise specified. All datasets were tested for normal distribution using Shapiro–Wilk tests. Skewed data were log‐transformed. Results were evaluated using unpaired (two‐sided) Student's *t*‐test, Welch's two‐sample *t‐*test, and one‐ or two‐way analysis of variance (ANOVA). The assessment of thermogenesis and indirect calorimetry were analyzed of the data with ANCOVA using weight as a covariable as reported.^[^ ^81^ ^]^ For correlation analyses, Pearson's correlation coefficient was used to analyze the correlation between CNNM4 mRNA levels and BMI, TNFα and IL1β mRNA levels. Statistically significant differences were considered to be *P < 0.05, **P < 0.01, ***P < 0.001, ****P < 0.0001. ns., not significant. The relevant statistical methods for each panel were detailed in the figure legend. All statistical analyses were performed using GraphPad or SPSS 22.0.

## Conflict of Interest

The authors declare no conflict of interest.

## Author Contributions

A.Z., J.J., C.Z., H.X., and W.Y. contributed equally to this work. W.W., X.Z., J.Z., and B.L. conceived and directed the study. A.Z., J.J., H.X., C.Z., W.Y., Z.N.Z, L.Y., Z.L., Y.D., C.F., X.W., and A.S. performed animal and cell experiments. H.L. and S.C. collected and organized clinical data. H.X. and H.F. performed scRNA‐seq downstream analysis. J.N. and W.W. contributed materials. A.Z., J.J., and W.W. wrote the manuscript, and X.Z., J.Z., and B.L. supervised the project. All authors provided input and reviewed the manuscript.

## Supporting information



Supporting Information

Supporting Information

## Data Availability

The data that support the findings of this study are available from the corresponding author upon reasonable request.
