# Supplementary material for: Thermogenic Adipocytes Promote M2 Macrophage Polarization through CNNM4‐Mediated Mg Secretion
Source: Adv Sci (Weinh). 2024 Nov 8;11(47):2401140. doi: 10.1002/advs.202401140 (PMC11653670; doi:10.1002/advs.202401140)
Supplement: Supplementary file 1 — Supporting Information [file ADVS-11-2401140-s002.docx]

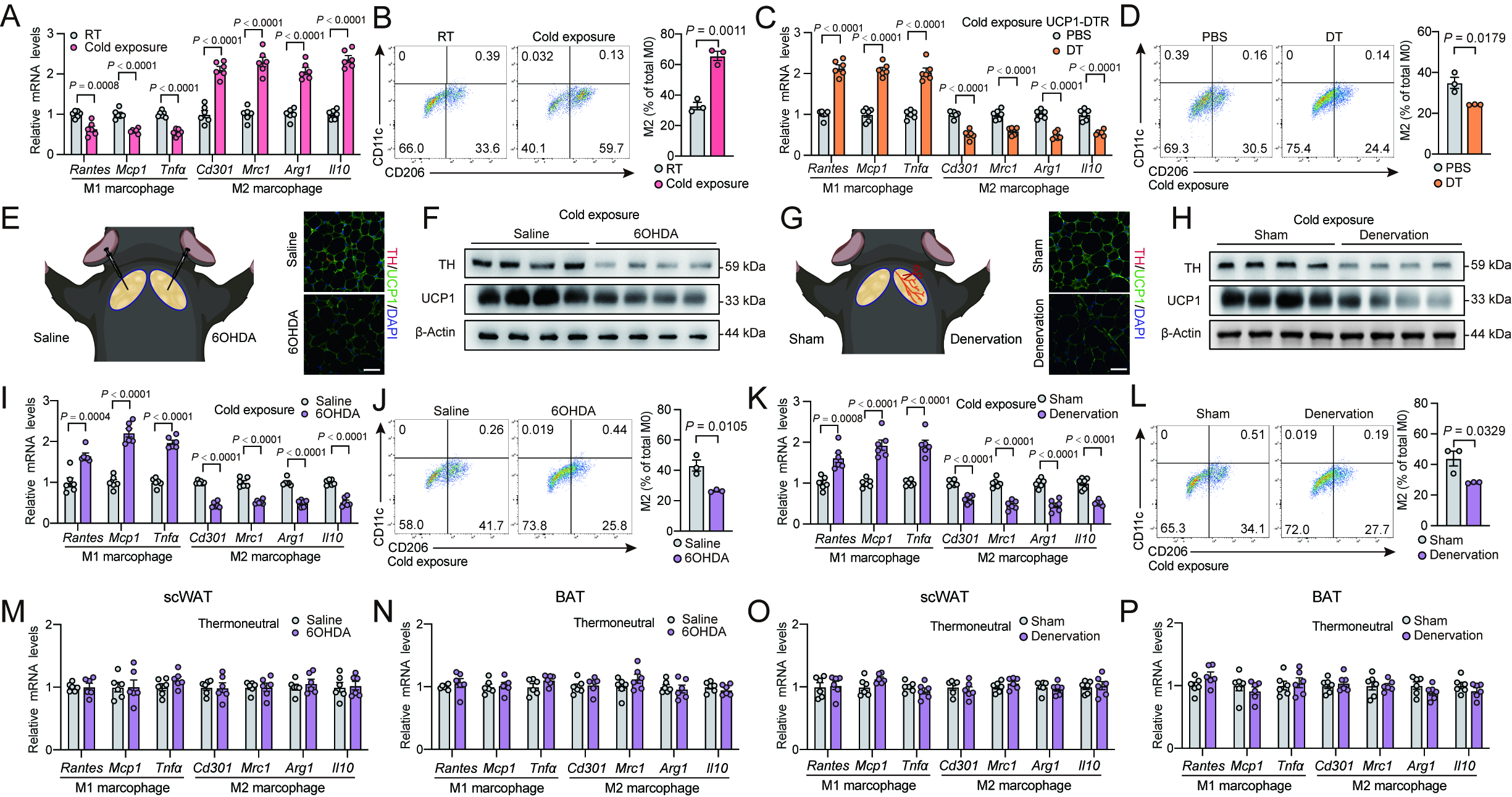


**Figure S1. Thermogenic adipocytes promote M2 macropage polarization in BAT.**

(A and B) Representative M1 and M2 macrophage marker gene expression (A) (n = 6) and flow cytometric plots and quantification demonstrate the numbers of M2 macrophages (CD206^+^/CD11c^−^) (B) (n = 3) in BAT from C57BL/6 mice under room temperature (25℃) or cold exposure (4℃);

(C and D) Representative M1 and M2 macrophage marker gene expression (C) (n = 6) and flow cytometric plots and quantification demonstrate the numbers of M2 macrophages (CD206^+^/CD11c^−^) (D) (n = 3) in BAT from UCP1-DTR mice injected with DT (200 ng/mice/day) or PBS;

(E-L) Representative TH and UCP1 immunostaining in BAT (E and G), UCP1and TH protein expression (F and H) , representative M1 and M2 macrophage marker gene expression (I and K) (n = 6) and flow cytometric plots and quantification demonstrate the numbers of M2 macrophages (CD206^+^/CD11c^−^) (J and L) (n = 3) in BAT of mice with 6-OHDA injection (E, F, I and J) or surgical transection of sympathetic neuron fibers (G, H, K and L) under cold exposure. Scale bar, 50 μm.

Data were expressed as means ± SEM. A-D and I-L were calculated by unpaired two-tailed Student’s t test.


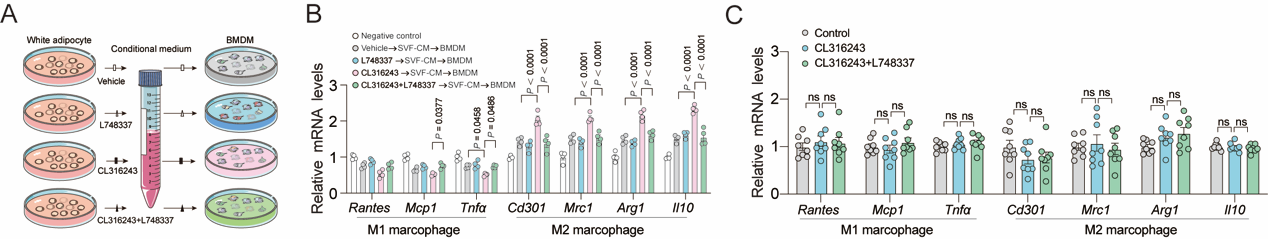


**Figure S2. The effect of white adipocyte conditioned medium (CM)** **on M2 macrophage polarization.**

(A) Scheme illustrating experimental set-up;

(B) Representative M1 and M2 macrophage marker gene expression in BMDMs stimulated with CM from unstimulated white adipocytes (vehicle) or CL316243, L748337, CL316243+L748337 treated white adipocytes (n = 4);

(C) Representative M1 and M2 macrophage marker gene expression in BMDMs stimulated with vehicle, CL316243 or CL316243+L74833 (n = 8).

Data were expressed as means ± SEM. B and C were calculated by two-way ANOVA followed with Bonferroni’s multiple comparison test.


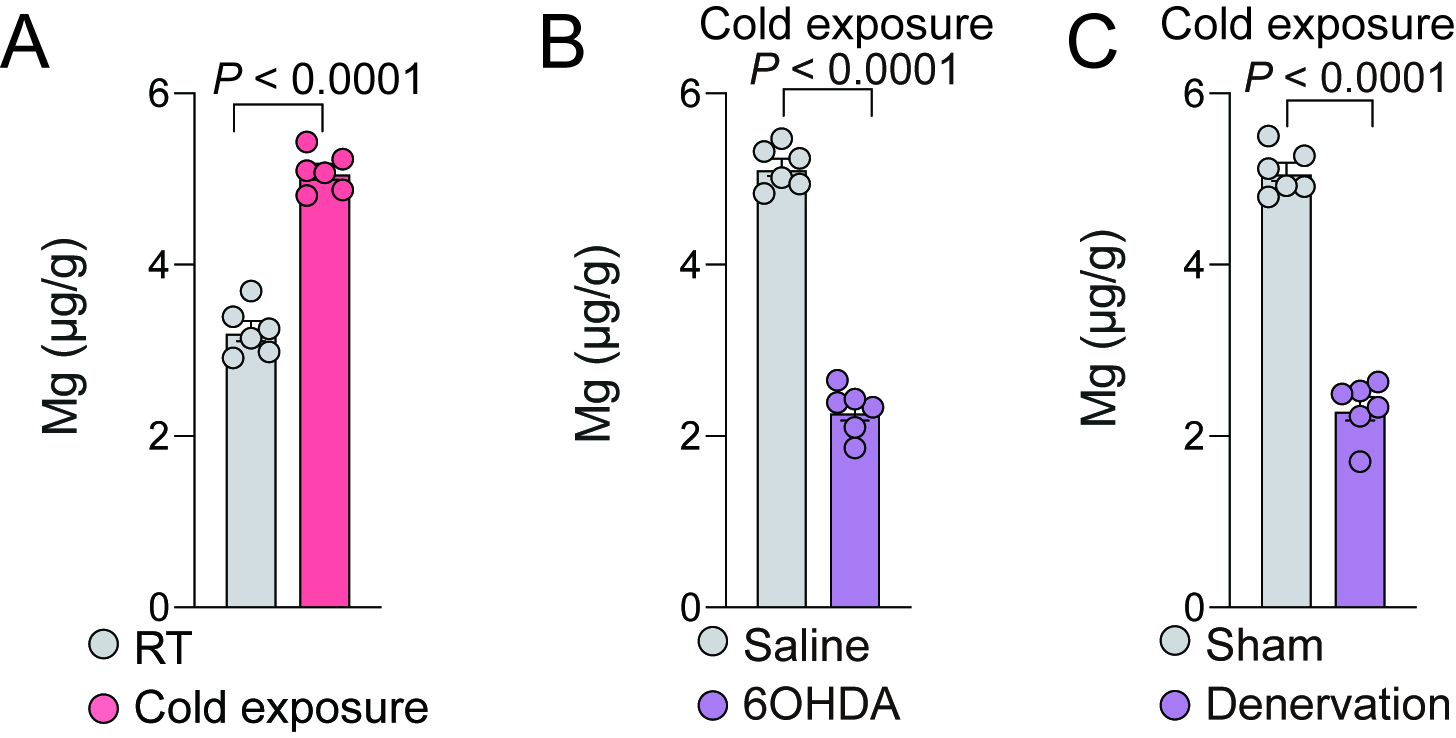


**Figure S3. Mg^2+^ level in BAT-derived interstitial fluid.**

Mg^2+^ level in BAT-derived interstitial fluid from C57BL6 mice under room temperature (25℃) or cold exposure (4℃) (n = 6) (A); sympathetic denervation achieved mice by local injection of 6-OHDA (5 mg/kg) (B) or surgical transection (C).

Data were expressed as means ± SEM. A-C were calculated by unpaired two-tailed Student’s t test.

**
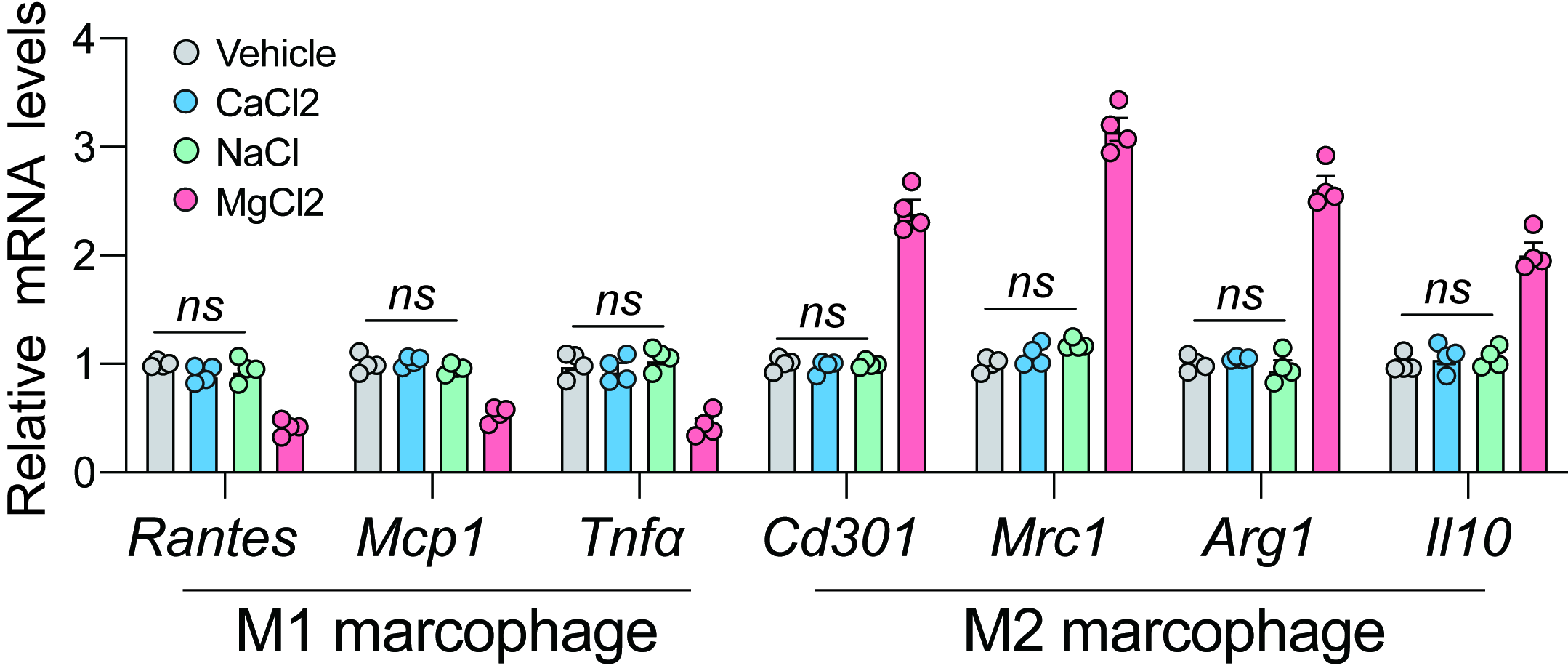
**

**Figure S4. Mg^2+^ but not Ca^2+^, Na^+^, and Cl^-^ promotes M2 macrophages polarization.**

Representative M1 and M2 macrophage marker gene expression (n = 4) in BMDMs with an equivalent concentration of Ca^2+^, Na^+^, and Cl^-^ (CaCl_2_: 5mM, NaCl: 10mM, or MgCl_2_: 5mM). Data were calculated by two-way ANOVA followed with Bonferroni’s multiple comparison test.


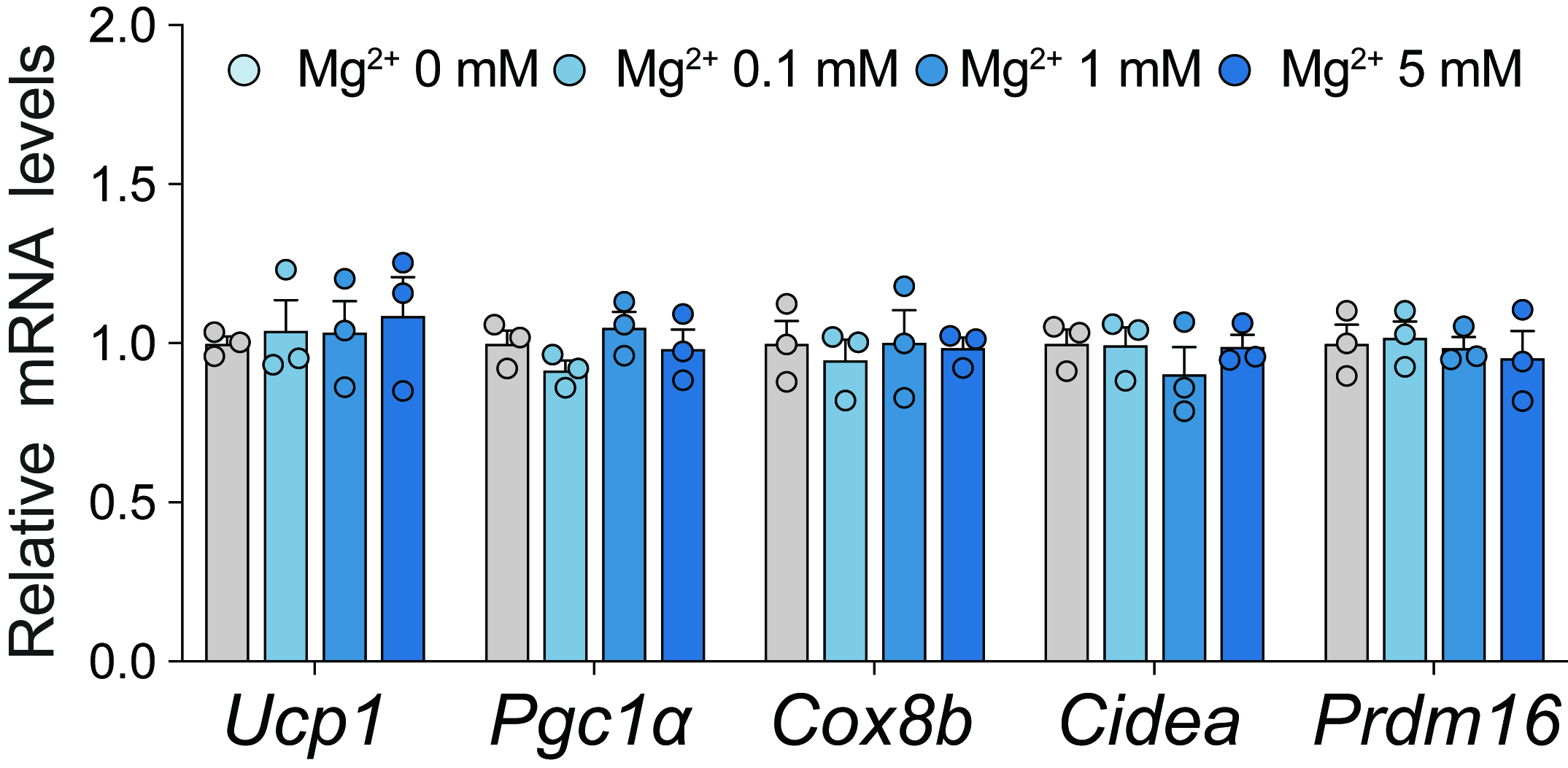


**Figure S5. Mg does not influence thermogenic genes expression in primary beige adipocytes.**

Representative thermogenic gene expression in primary beige adipocytes after stimulation with indicated concentration of Mg (n = 3). Data were calculated by one-way ANOVA.


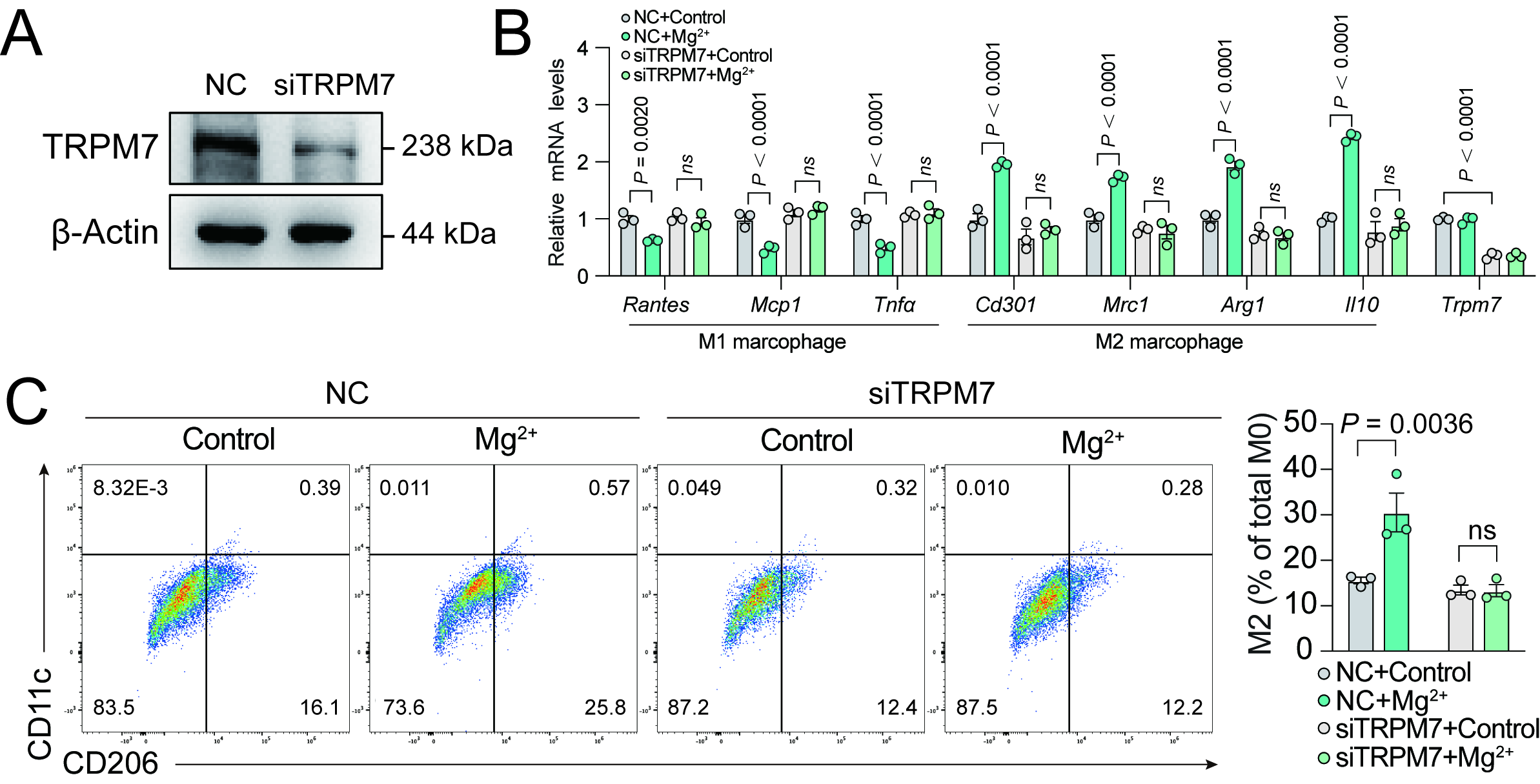


**Figure S6. TRPM7 promotes M2 macrophage polarization.**

(A) Representative immunoblots of TRPM7 expression in BMDMs after 72-hour transfection. (B and C) Representative flow cytometric plots and quantification demonstrate the numbers of M2 macrophages (CD206^+^/CD11c^−^) (B) (n = 3) and M1 and M2 macrophage marker gene expression (C) (n = 3) in siTRPM7 or control-transfected BMDMs after stimulation with MgCl_2_ (5 mM).

Data were expressed as means ± SEM. B and C were calculated by two-way ANOVA followed with Bonferroni’s multiple comparison test.


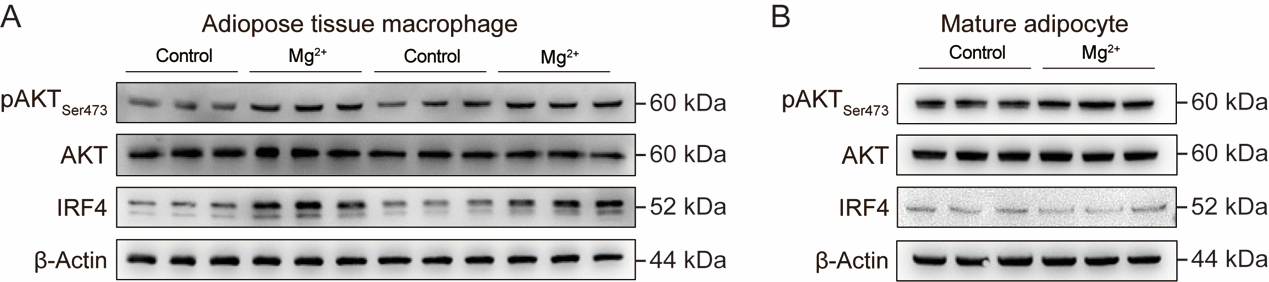


**Figure S7. MgCl2 stimulation of mTORC2/AKT phosphorylation in macrophages or mature adipocytes within adipose tissues.**

(A) Representative immunoblots showing mTORC2 mediated AKT phosphorylation and IRF4 expression in MACS-isolated adipose tissue macrophages from mice locally injected with MgCl2 in scWAT. (B) Representative immunoblots showing mTORC2 mediated AKT phosphorylation in mature adipocytes from mice locally injected with MgCl2 in scWAT.


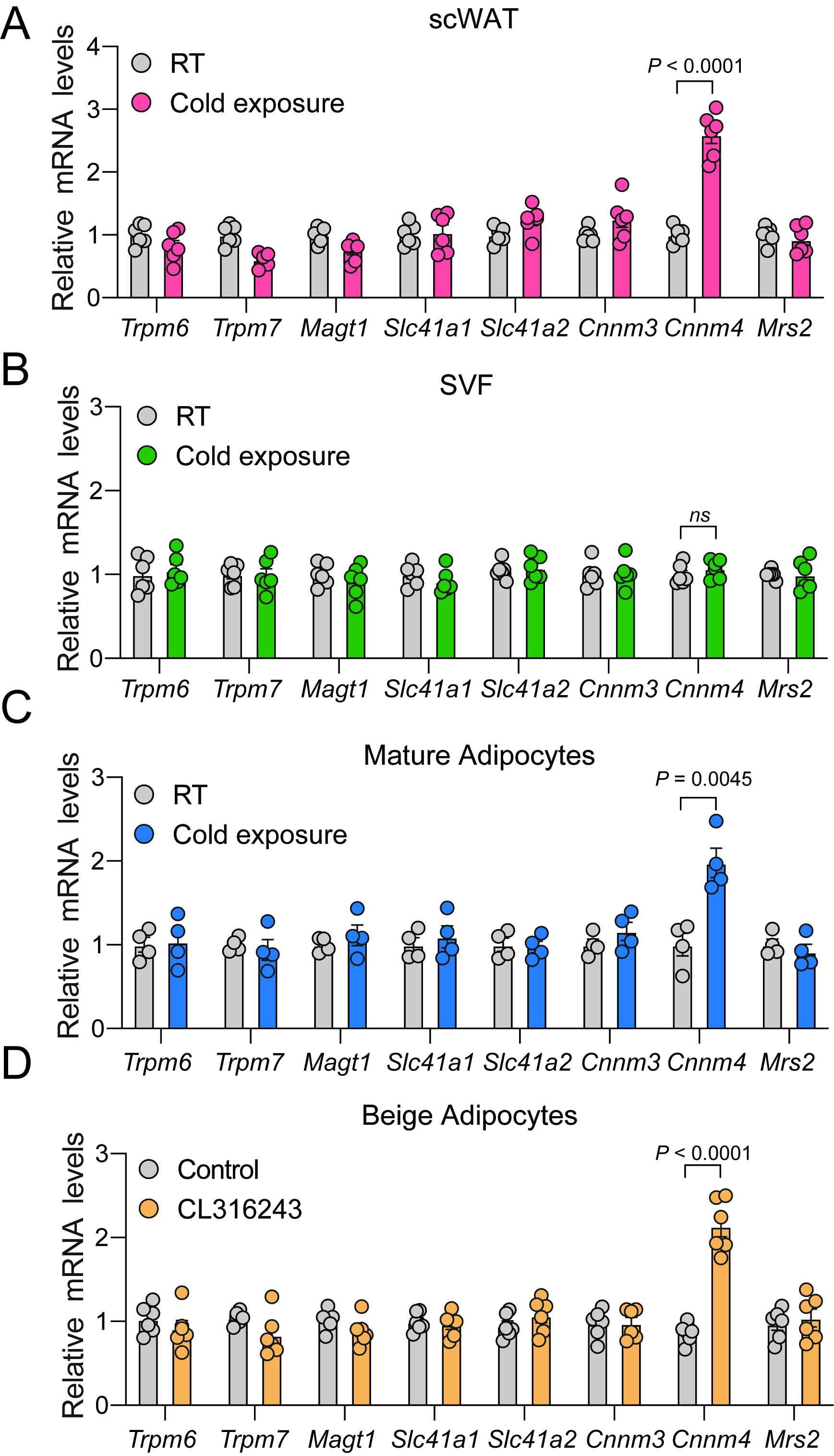


**Figure S8. Cold induces CNNM4 expression in adipocytes.**

(A-C) Representative Mg transporters (*Mrs2, Cnnm4, Cnnm3, Slc41a1, Slc41a2, Magt1, Trpm6,* and *Trpm7*) gene expression in scWAT (A) (n =6), SVF (B) (n =6), or mature adipocyte (C) (n =4) of C57BL/6 mice under cold exposure;

(D) Representative Mg transporters (*Mrs2, Cnnm4, Cnnm3, Slc41a1, Slc41a2, Magt1, Trpm6,* and *Trpm7*) gene expression in primary beige adipocytes after CL316243 stimulation (n = 6).

Data were expressed as means ± SEM. A-D was calculated by unpaired two-tailed Student’s t test.

**
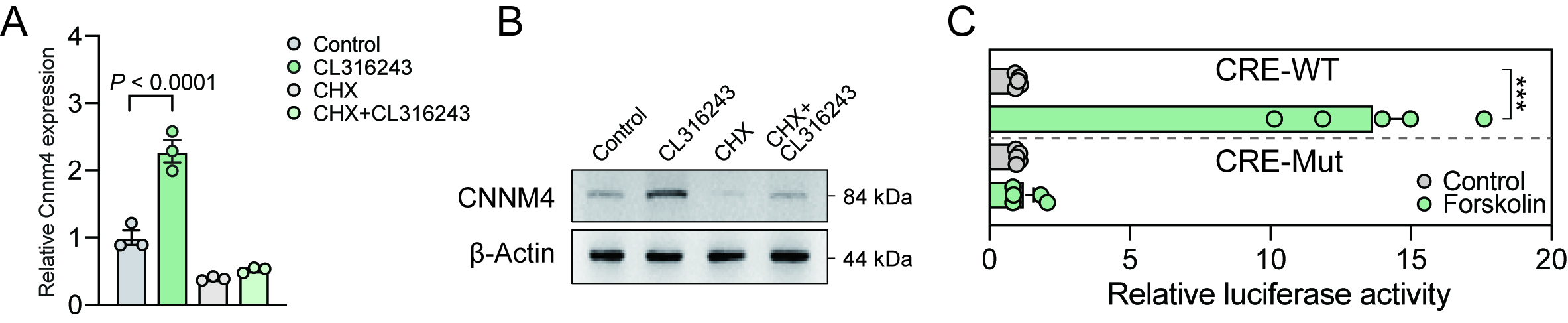
**

**Figure S9. CNNM4 is the primary transcriptional target of CREB.**

(A and B) CNNM4 mRNA levels (A) (n = 3) and protein levels (B) in primary beige adipocytes treated with cycloheximide (CHX) and CL316243;

(C) Effect of Fosklin (5 μM) on CNNM4 WT or CRE sites mutated promoter luciferase reporter activity in HEK293T cells (n = 5).

Data were expressed as means ± SEM. A and C were calculated by two-way ANOVA followed with Bonferroni’s multiple comparison test.


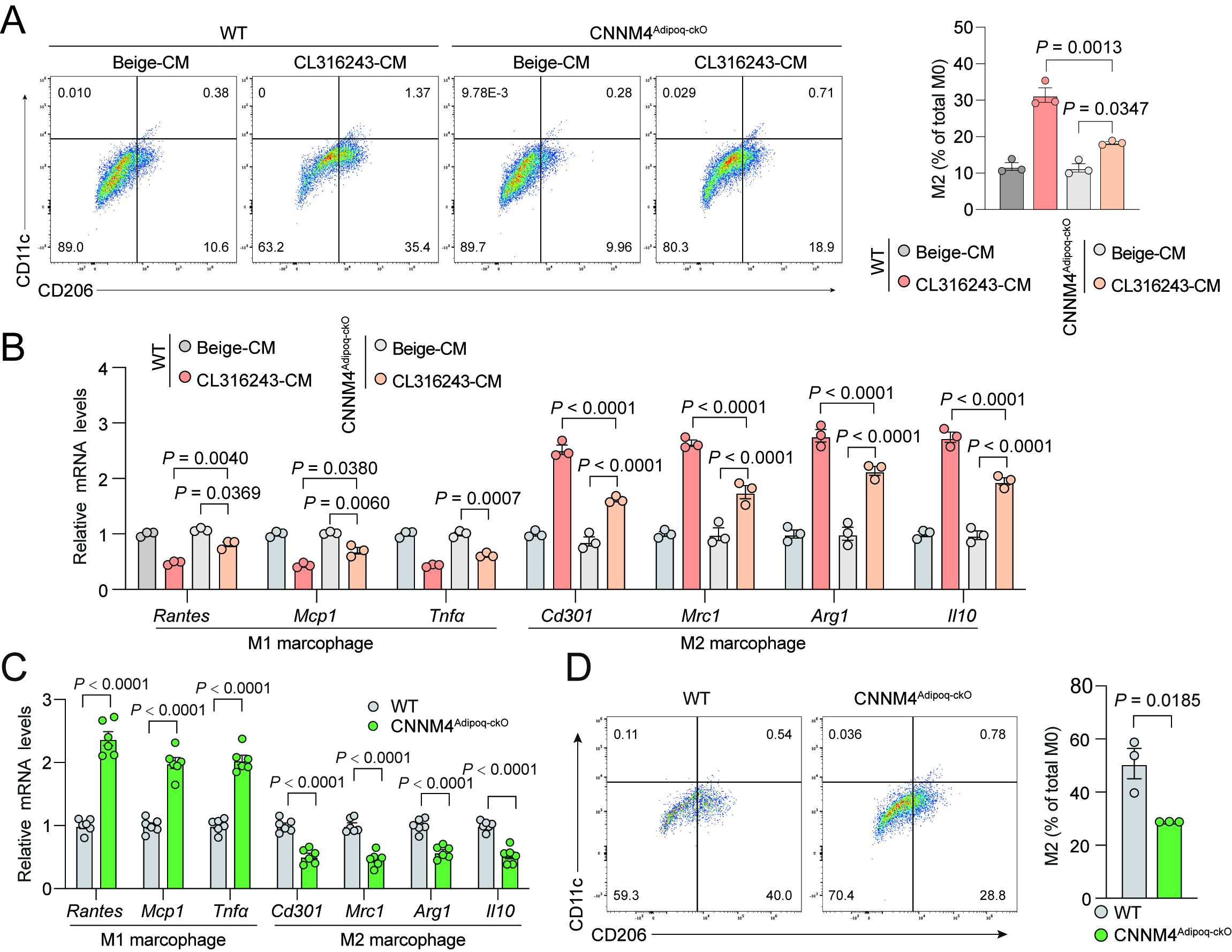


**Figure S10. Loss of CNNM4 in beige adipocytes impaires M2 macrophage polarization.**

(A and B) Representative flow cytometric plots and quantification demonstrate the numbers of M2 macrophages (CD206^+^/CD11c^−^) (A) (n = 3) and M1 and M2 macrophage marker gene expression (B) (n = 3) in BMDMs stimulated with CM from CNNM4^Adipoq-cKO^ or WT beige adipocytes treated with CL316243;

(C and D) Representative M1 and M2 macrophage marker gene expression (C) (n = 6) and flow cytometric plots and quantification demonstrate the numbers of M2 macrophages (CD206^+^/CD11c^−^) (D) (n = 3) in BAT from CNNM4^Adipoq-cKO^ or WT mice under cold exposure.

Data were expressed as means ± SEM. A and B were calculated by two-way ANOVA followed with Bonferroni’s multiple comparison test; C and D were calculated by unpaired two-tailed Student’s t test.


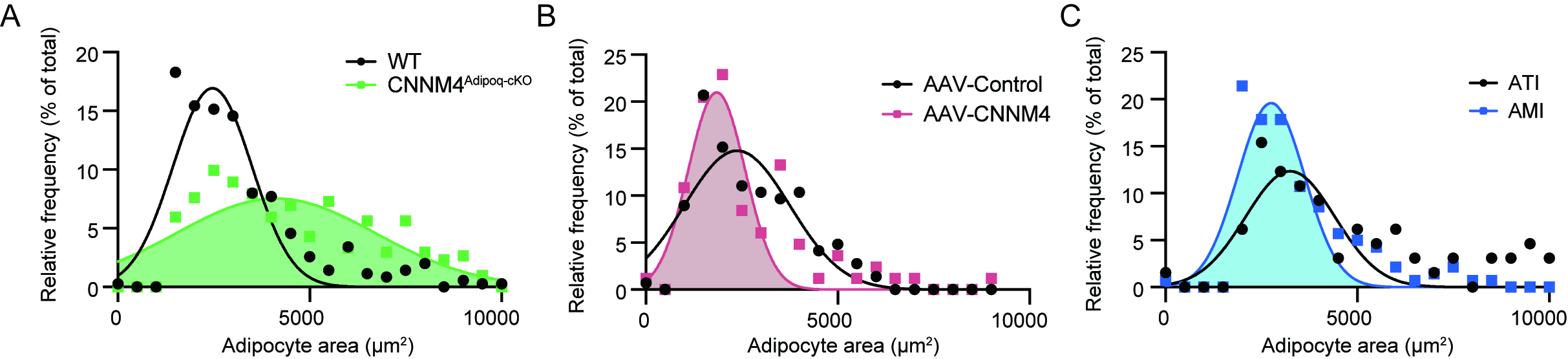


Figure S11. Relative frequencies and present adipocyte area distribution of adipose tissue from CNNM4^Adipoq-cko^, CNNM4-overexpressed, and AMI mice.


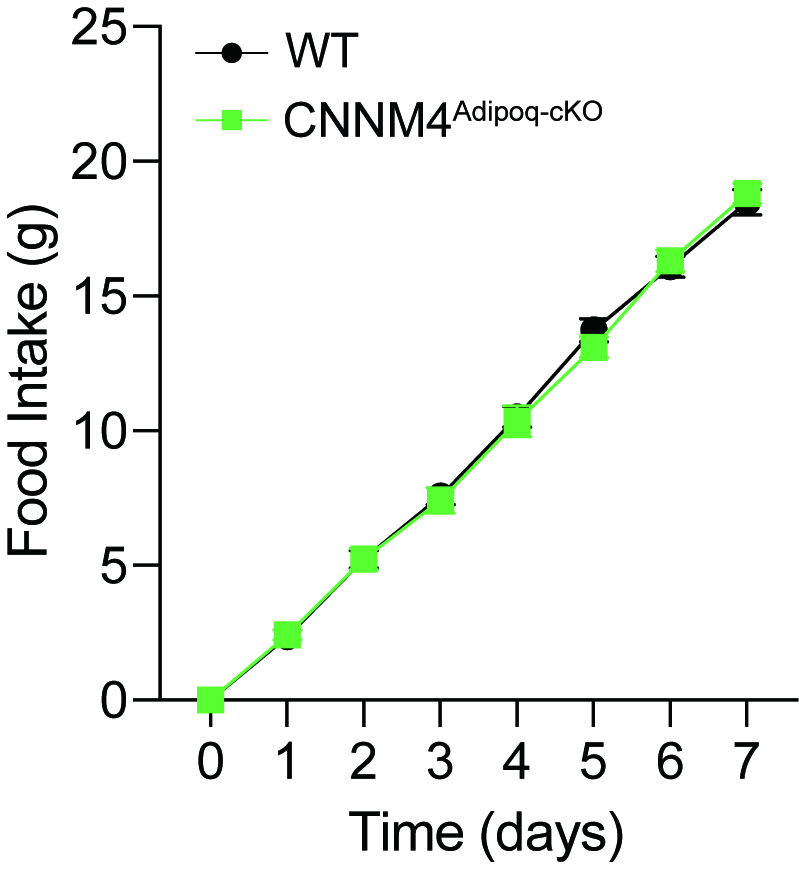


**Figure S12. Food intake of WT and CNNM4 ^Adipoq-cKO^ mice.**

Food intake of CNNM4^Adipoq-cKO^ or WT mice under RD feeding (n = 6).

Data were expressed as means ± SEM. Data was calculated by two-way ANOVA followed with Bonferroni’s multiple comparison test.


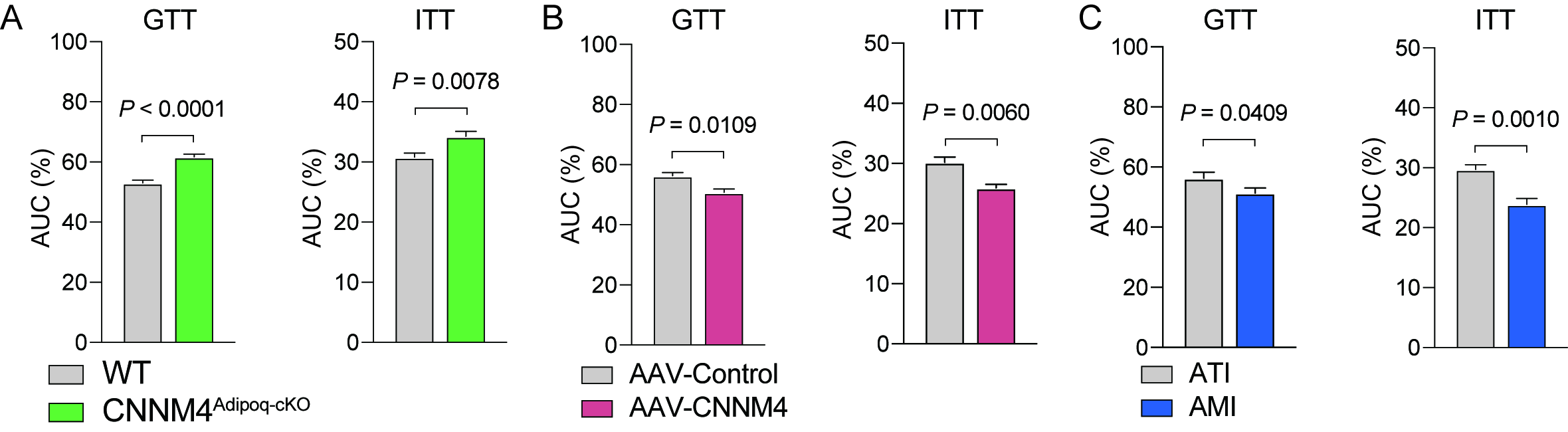


**Figure S13. AUC of GTT or ITT.**

Data were expressed as means ± SEM. Data was calculated by unpaired student’s t test.


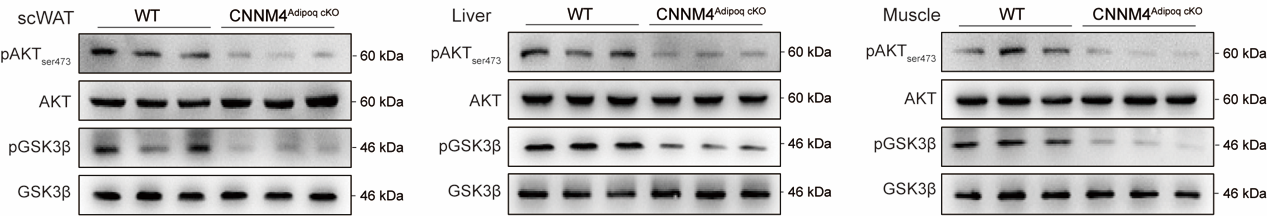


**Figure S14. CNNM4 ^Adipoq-cKO^ mice show impaired insulin signaling in scWAT, liver and muscle**

Representative immunoblots showing effects of CNNM4^Adipoq-cKO^ on insulin signaling in scWAT, liver, and muscle tissues under room temperature.


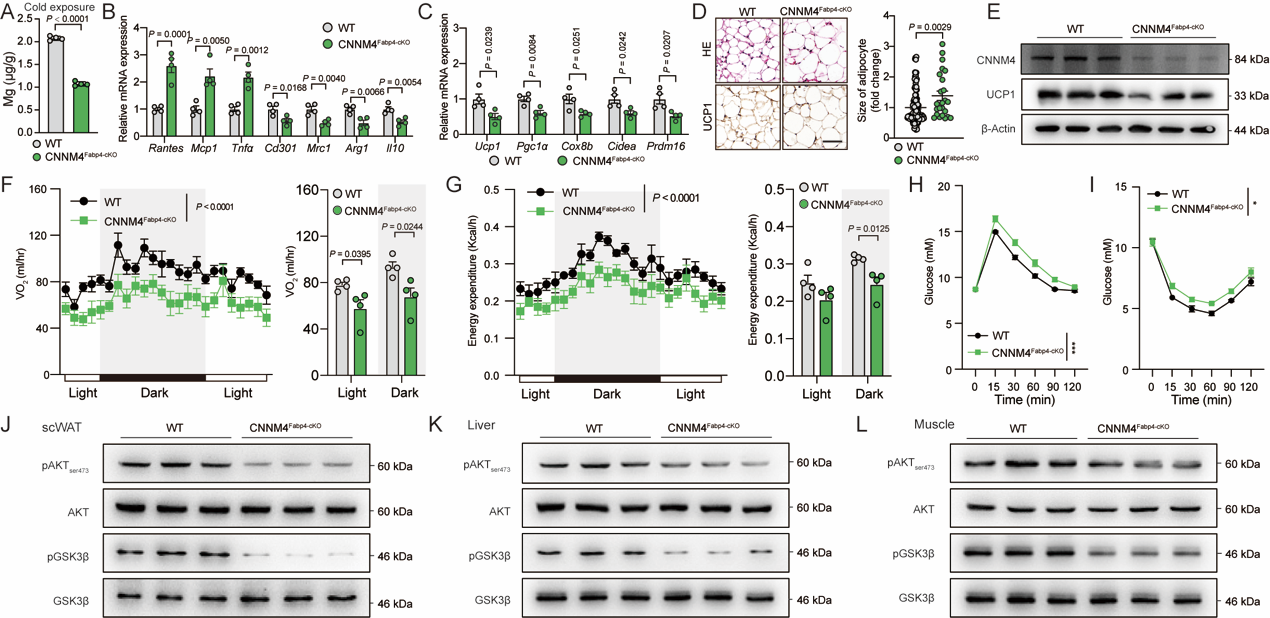


**Figure S15. CNNM4^Fabp4-cKO^ mice show impaied M2 macrophage polarization, thermogenesis and insulin sensitivity.**

(A) Mg levels in scWAT-derived interstitial fluid from CNNM4^Fabp4-cKO^ or WT mice under cold exposure (n = 4);

(B and C) Representative M2 macrophage marker gene expression (B) and thermogenic gene expression (C) in scWAT from CNNM4^Fabp4-cKO^ or WT mice under cold exposure (n = 4);

(D-G) Representative H&E staining (Top) and UCP1 immunohistochemistry (Down) (D), UCP1 and CNNM4 protein expression (E) in scWAT, and VO_2_ (F), energy expenditure (G) of CNNM4^Fabp4-cKO^ or WT mice under cold exposure (n = 4);

(H and I) Glucose tolerance tests (H) and insulin tolerance tests (I) of CNNM4^Fabp4-cKO^ or WT mice under room temperature (n = 4);

(J-L) Representative immunoblots showing insulin signaling of CNNM4^Fabp4-cKO^ or WT mice in scWAT (J), liver (K), and muscle (L) under room temperature.

Data were expressed as means ± SEM. A-D, F and G were calculated by unpaired two-tailed Student’s t test; F-I were calculated by two-way ANOVA followed with Bonferroni’s multiple comparison test; F and G were analyzed by ANCOVA with body weight as covariant.


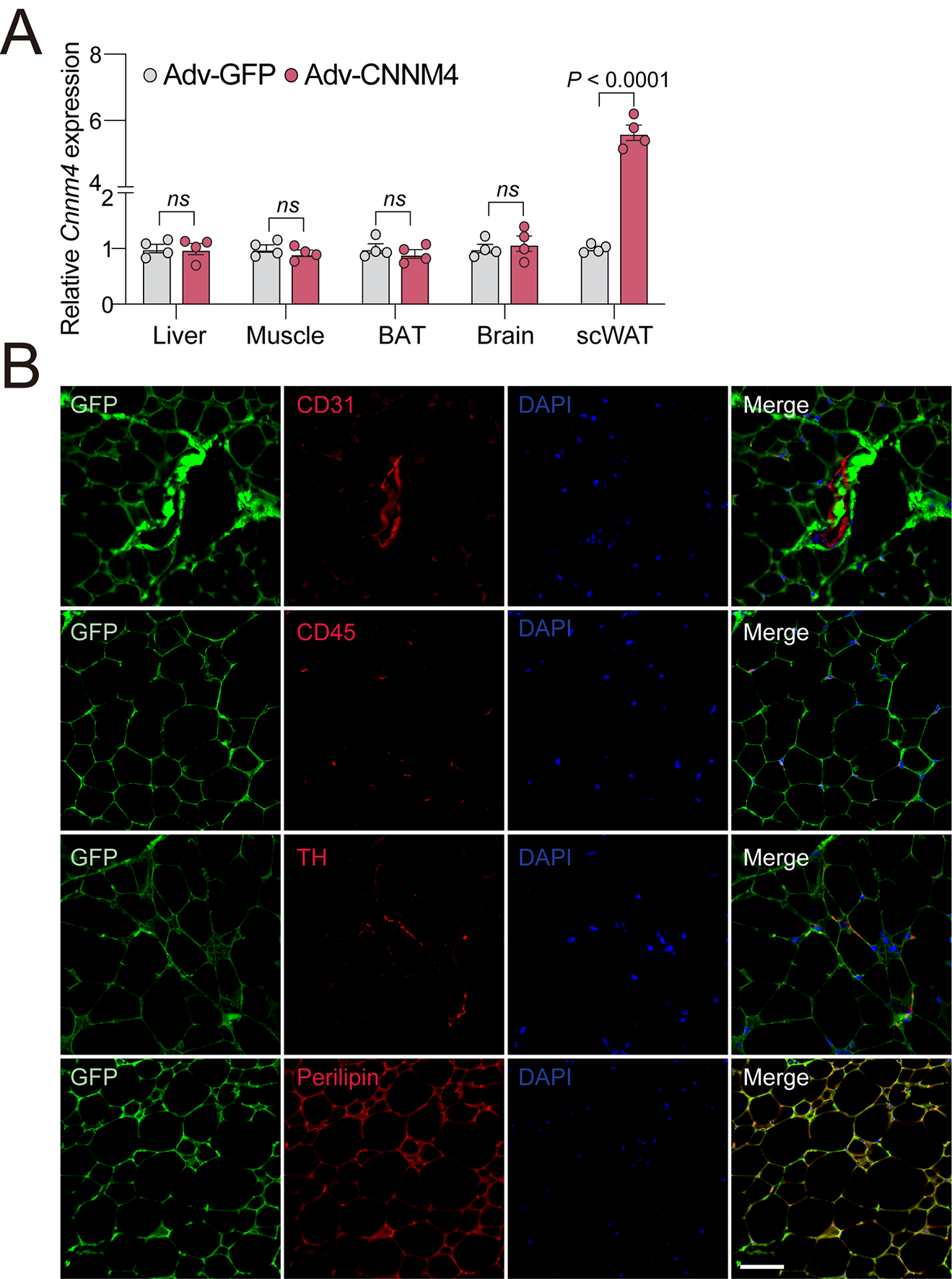


**Figure S16. Adv-CNNM4 infects adipocytes, but not sympathetic neurons, vessels, and immune cells.**

(A) CNNM4 mRNA levels in scWAT, BAT, liver, muscle and brain from Adv-GFP and Adv-CNNM4 injected mice (n = 4);

(B) Co-staining of adenovirus infected cells (GFP positive) with adipocytes (Perilipin positive), sympathetic neurons (TH positive), vessels (CD31 positive), and immune cells (CD45 positive). Scale bar, 50 μm.


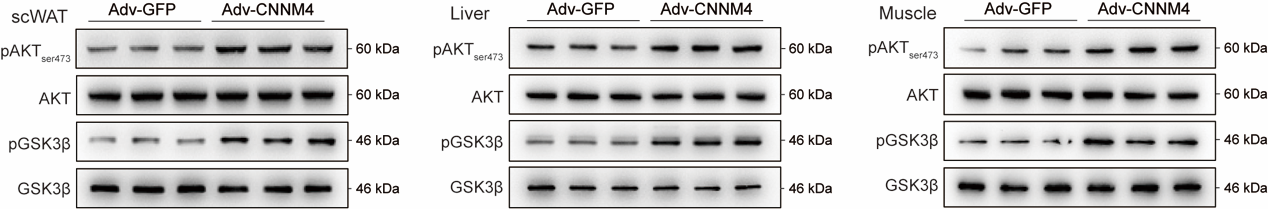


**Figure S17. Overexpression of CNNM4 in adipose tissue promotes insulin signaling**

Representative immunoblots showing effects of CNNM4 over-expression on insulin signaling in scWAT, liver, and muscle tissues under room temperature.


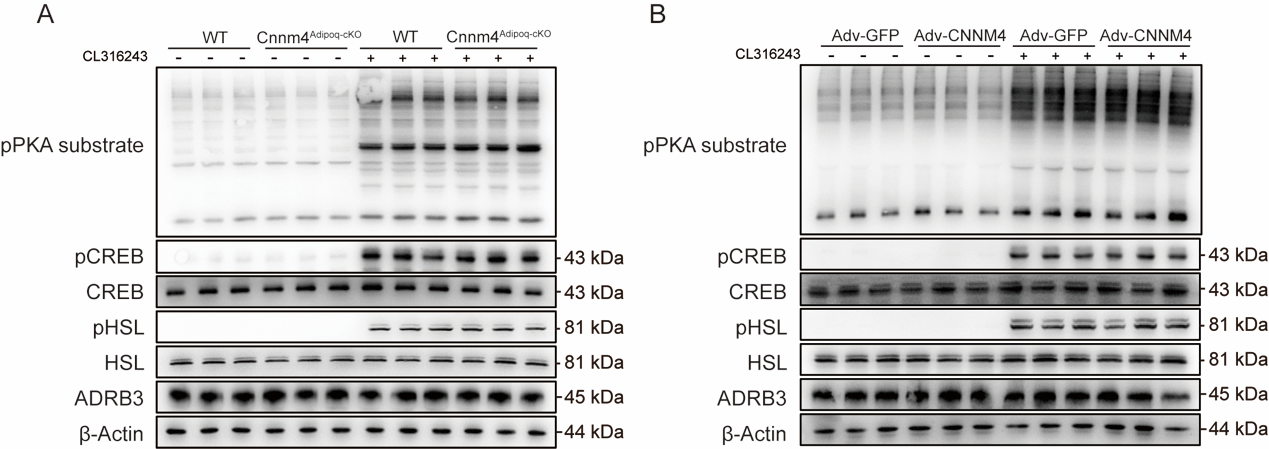


**Figure S18. CNNM4 does not influence β_3_AR activation and ADRB3 expression.**

(A and B) Representative immunoblots of β_3_AR signal pathway activation and ADRB3 expression in scWAT from Adv-CNNM4 (A) or CNNM4^Adipoq-cKO^ (B) mice after stimulation with CL316243.


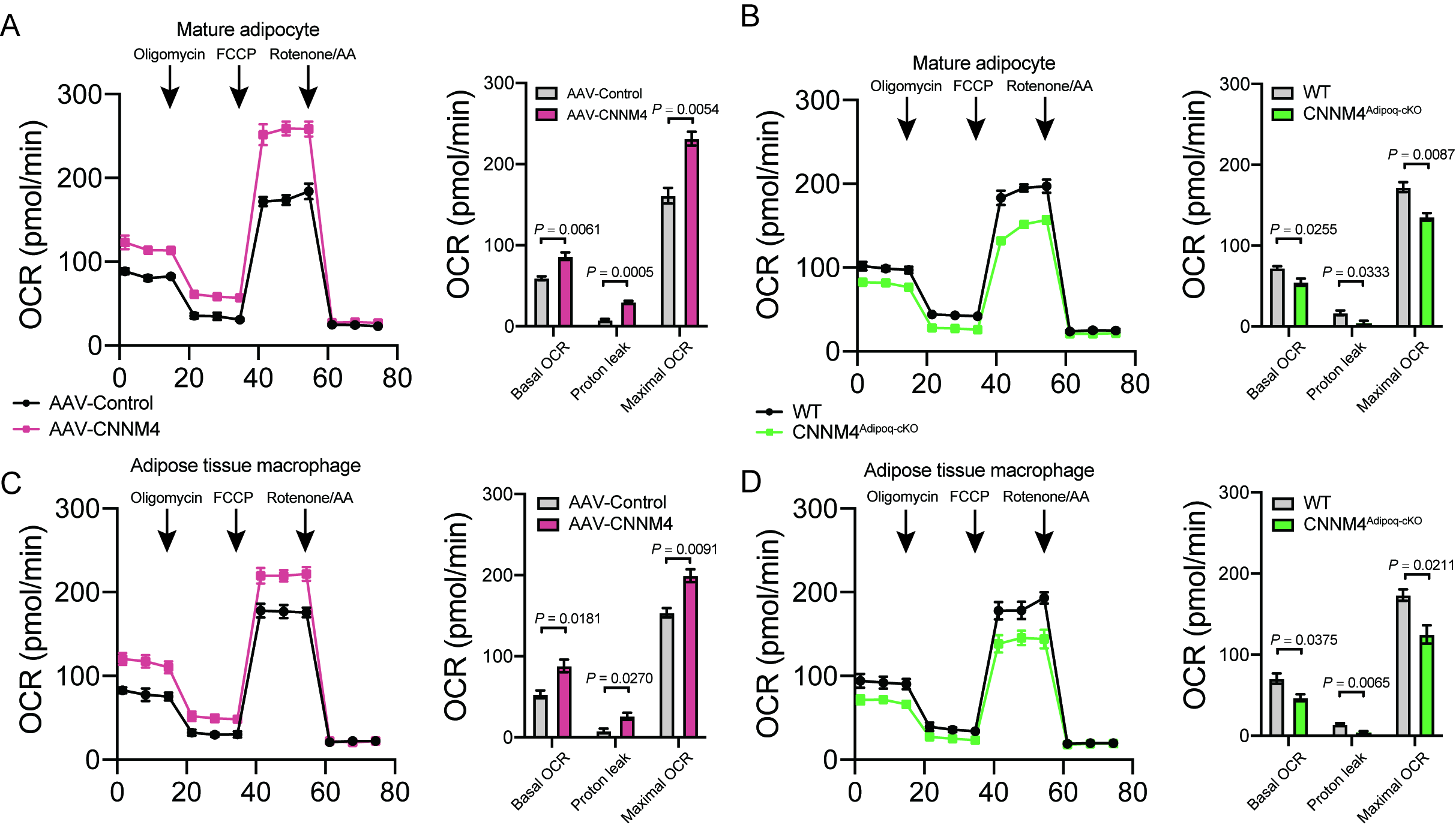


**Figure S19. Analysis of oxygen consumption in mature adipocytes and ATMs from CNNM4-KO or CNNM4-OE mice.**

Data were expressed as means ± SEM. A-D were calculated by A-C were calculated by unpaired two-tailed Student’s t test. n = 3 independently biological repeats.


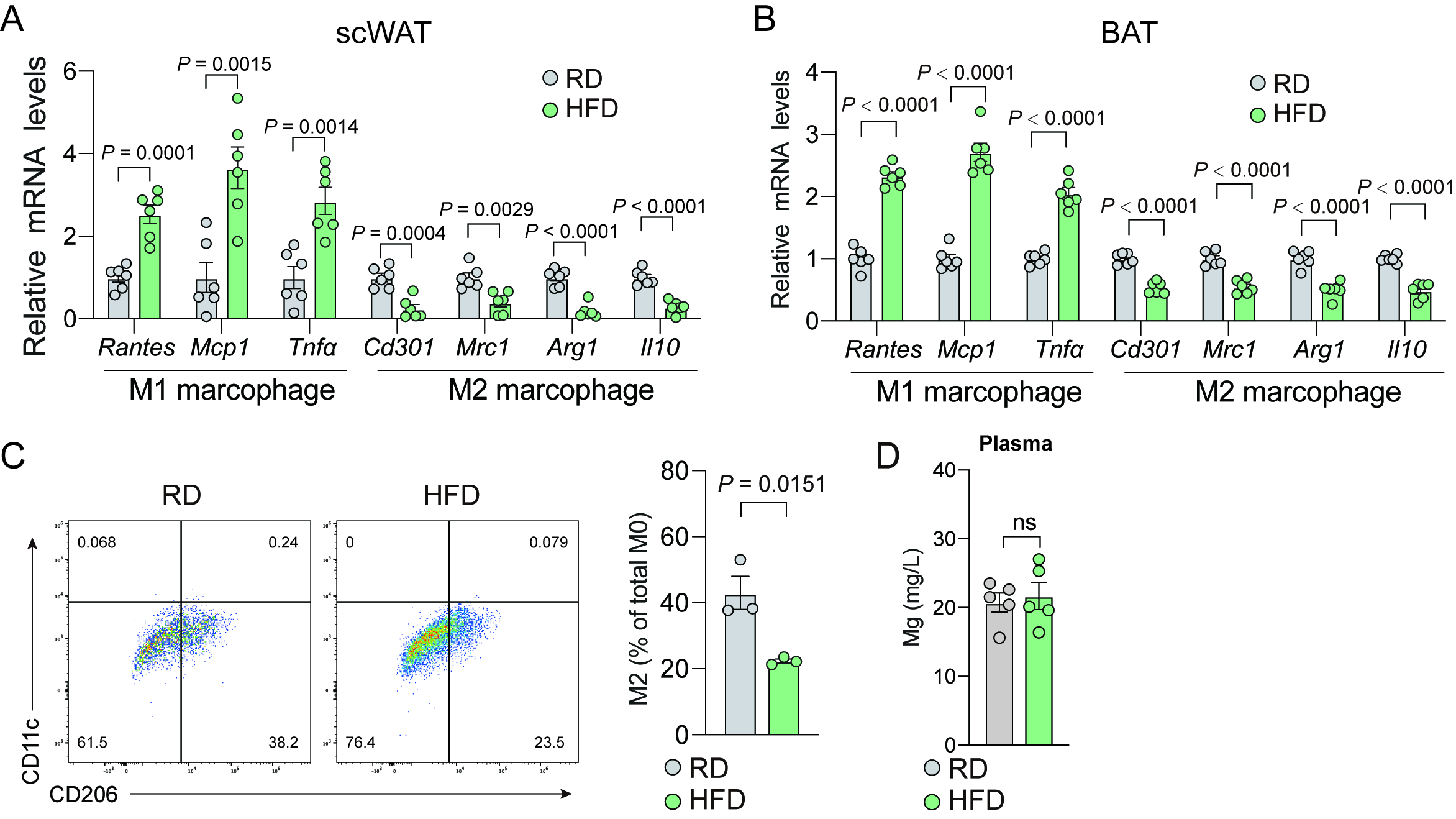


**Figure S20. Obesity suppresses M2 macrophage polarization.**

(A and B) Representative M1 and M2 macrophage marker gene expression in scWAT(A) and BAT (B) from mice fed with HFD or RD for 12 weeks (n = 6);

(C) Representative flow cytometry plots and quantification demonstrate the numbers of M2 macrophages (CD206+/CD11C−) in the BAT from mice fed with HFD or RD for 12 weeks (n = 3);

(D) Mg levels in plasma from mice fed with HFD or RD for 12 weeks (n = 5);

Data were expressed as means ± SEM. A-D were calculated by unpaired two-tailed Student’s t test.


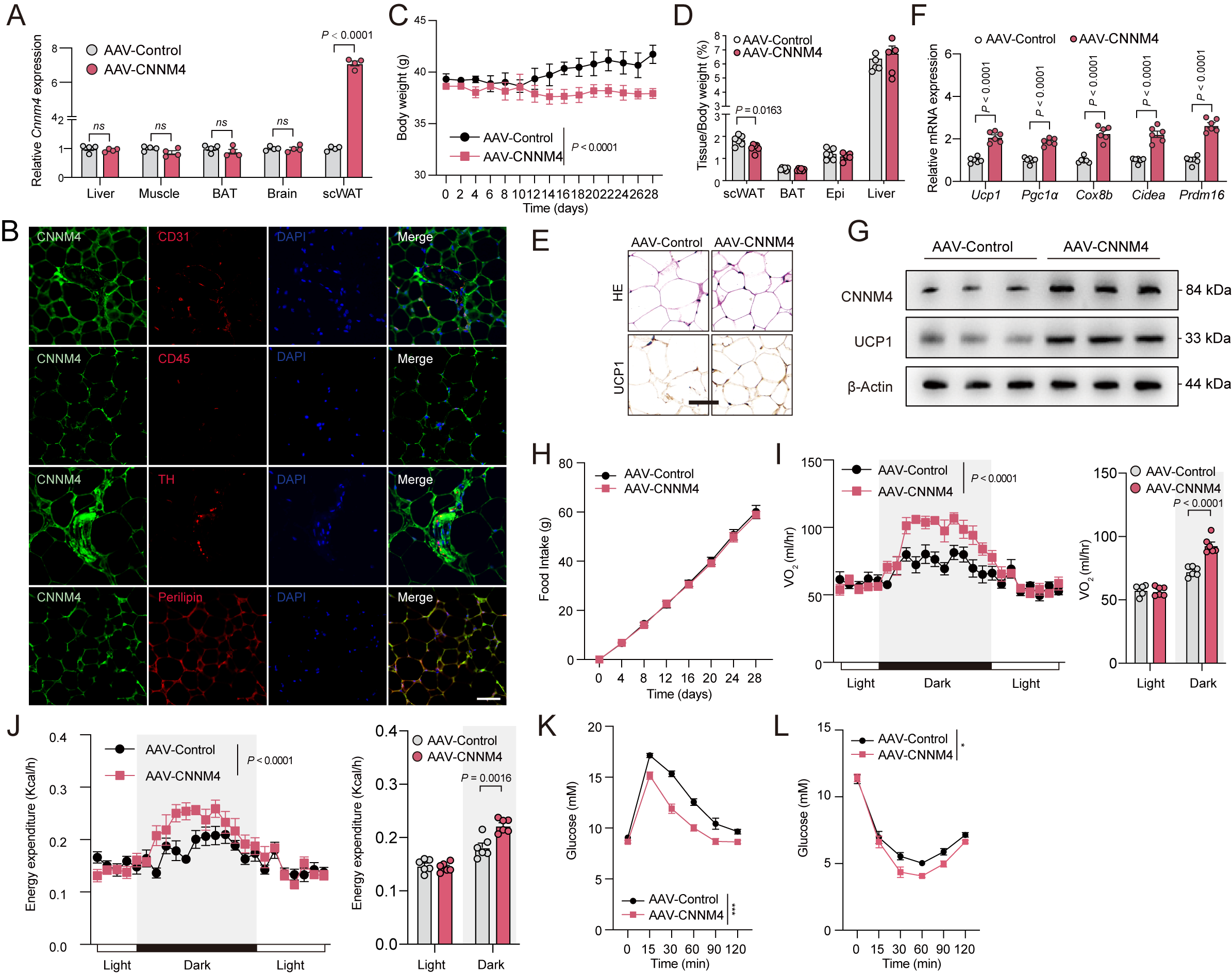


**Figure S21. Anti-obesity effects of CNNM4.**

(A) CNNM4 mRNA levels in liver, muscle, BAT, scWAT and brain tissues after AAV local injection (n = 4);

(B) Co-staining of adenovirus infected cells (CNNM4 positive) with adipocytes (Perilipin positive), sympathetic neurons (TH positive), vessels (CD31 positive), and immune cells (CD45 positive). Scale bar, 50 μm;

(C) Body weight and (D) indicated tissue weight of HFD-fed mice injected with AAV-CNNM4 for 4 weeks (n = 6);

(E-J) Representative H&E staining (Top) and UCP1 immunohistochemistry (Down) (E), thermogenic genes expression (F), UCP1 and CNNM4 protein expression (G) in scWAT, food intake (H), VO_2_ (I), and energy expenditure (J) of HFD-fed mice injected with AAV-CNNM4 for 4 weeks (n = 6);

(K and L) Glucose tolerance tests (K) and insulin tolerance tests (L) of HFD-fed mice injected with AAV-CNNM4 for 4 weeks (n = 6). A, D, F, I, and J were calculated by unpaired two-tailed Student’s t test; C, K, and L were calculated by two-way ANOVA with Bonferroni’s multiple comparison test; I and J were analyzed by ANCOVA with body weight as covariant.

**
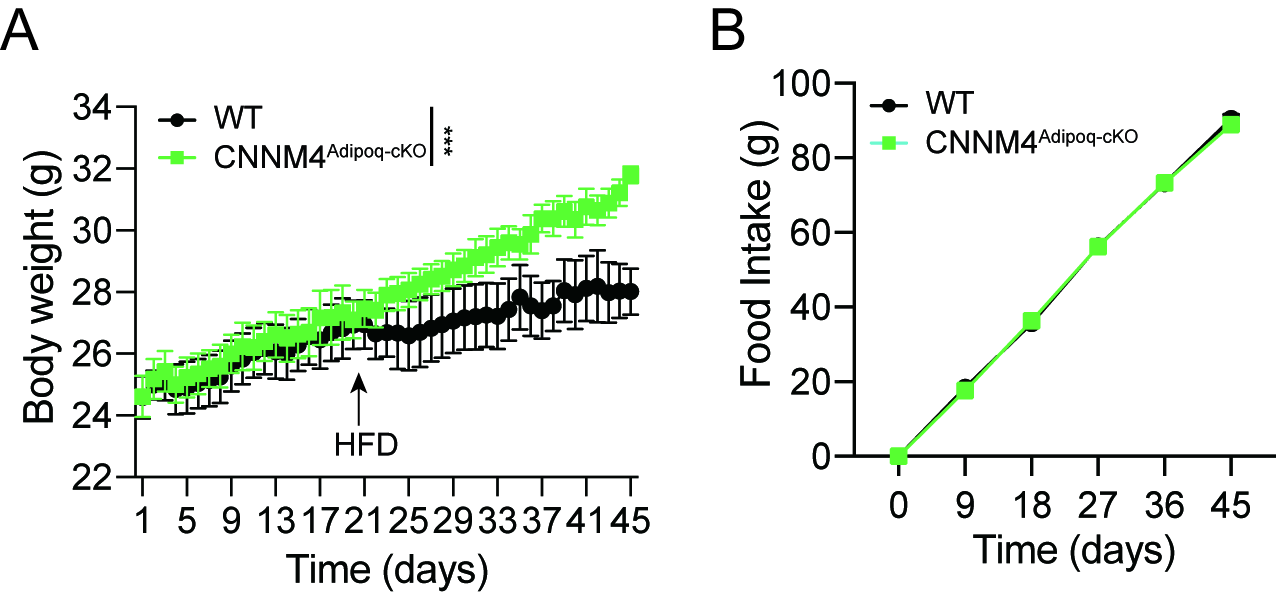
**

**Figure S22. Loss of CNNM4 in adipose tissue deteriorates obesity.**

(A) Body weight and (B) food intake of CNNM4^Adipoq-cKO^ or WT mice under RD or HFD feeding (n = 6). Data were expressed as means ± SEM. A and B were calculated by two-way ANOVA with Bonferroni’s multiple comparison test.

_
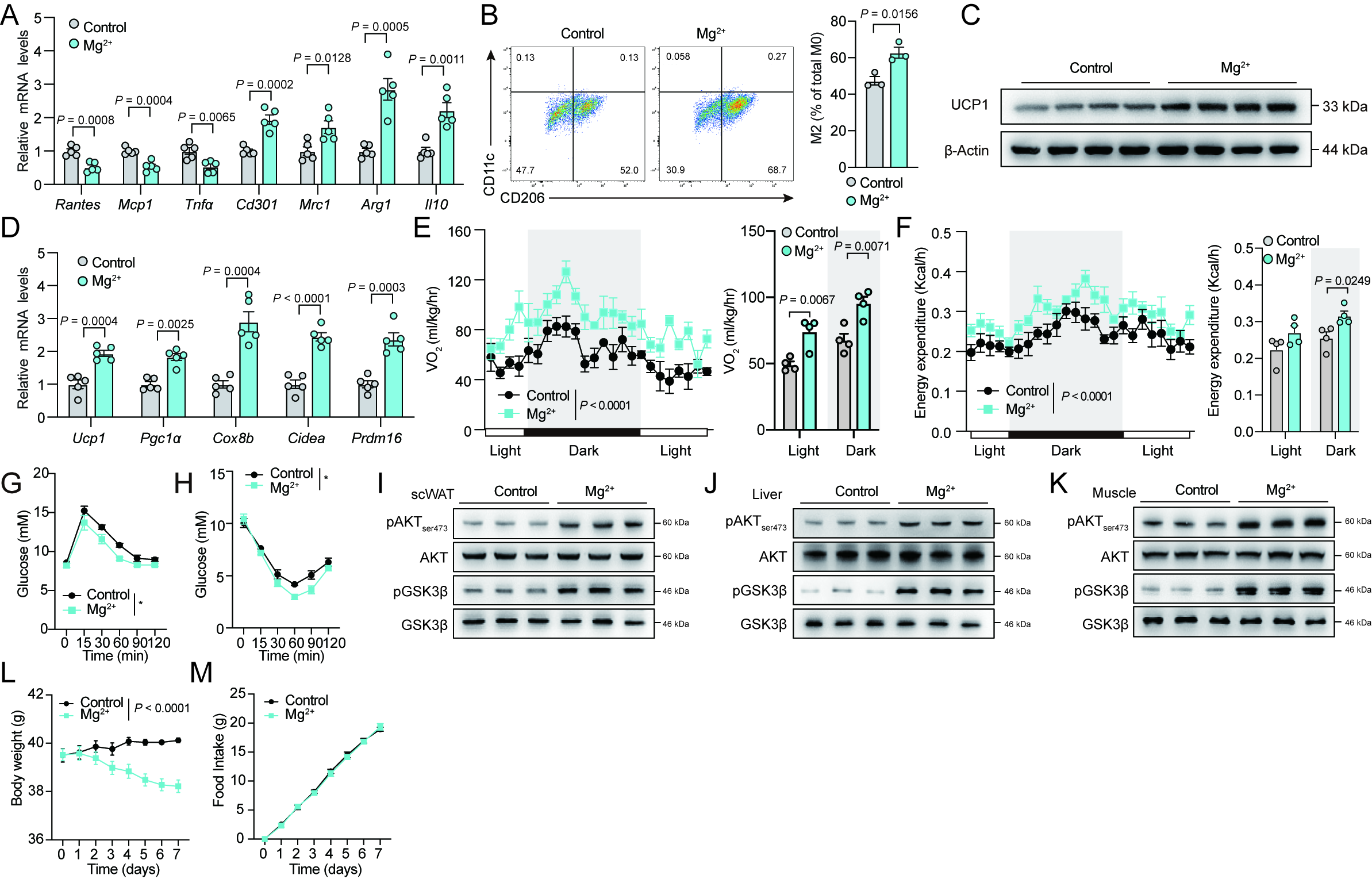
_

**Figure S23. Mg supplement promotes thermogenesis, insulin sensitivity and anti-obesity.**

(A and B) Representative M1 and M2 macrophage marker gene expression (A) (n = 5) and flow cytometric plots and quantification demonstrate the numbers of M2 macrophages (CD206^+^/CD11c^−^) (B) (n = 3) in scWAT from HFD-fed mice with local injection of MgCl_2_;

(C-M) UCP1 protein levels (C), representative thermogenic gene expression (D) (n = 5) in scWAT, VO_2_ (E), energy expenditure (F), glucose tolerance tests (G), insulin tolerance tests (H), representative immunoblots of insulin signaling in scWAT (I), liver (J), and muscle tissues (K), body weight (L) and food intake (M) of HFD-fed mice with local injection of MgCl_2_ (n = 4).

Data were expressed as means ± SEM. A, B, and D-F were calculated by unpaired two-tailed Student’s t test; G, H, L, and M were calculated by two-way ANOVA with Bonferroni’s multiple comparison test A; E and F were analyzed by ANCOVA with body weight as covariant.


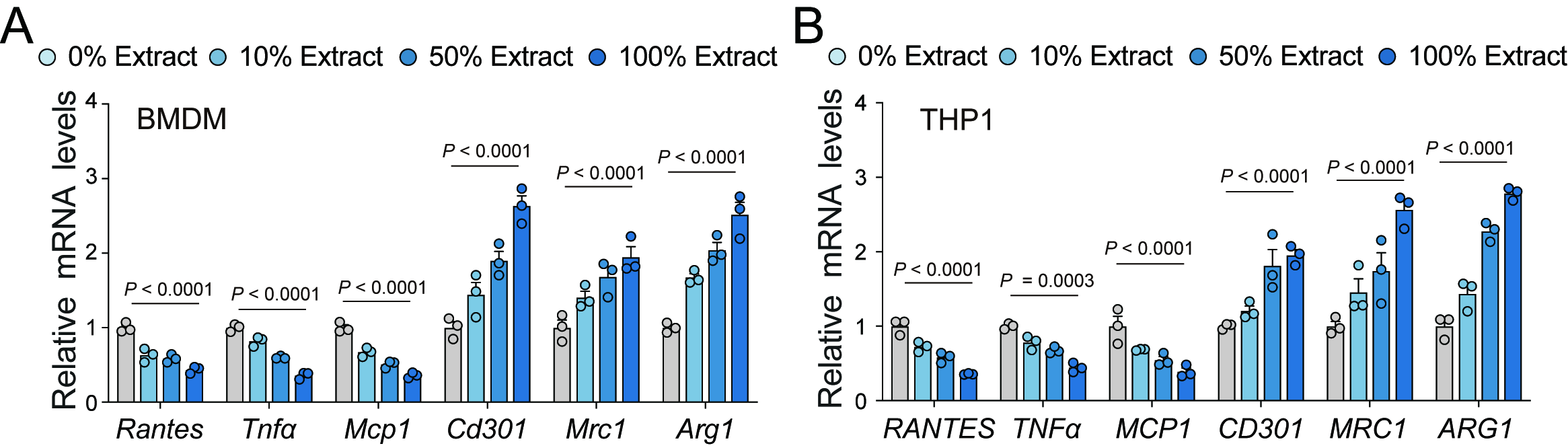


**Figure S24. AMI promotes M2 macrophage polarization *in vitro*.**

(A and B) Representative M1 and M2 macrophage marker gene expression in BMDMs (A) and THP1 cells (B) after stimulation with the indicated concentration of Mg wire-derived extract (n = 3).

Data were expressed as means ± SEM. A and B were calculated by one way ANOVA.


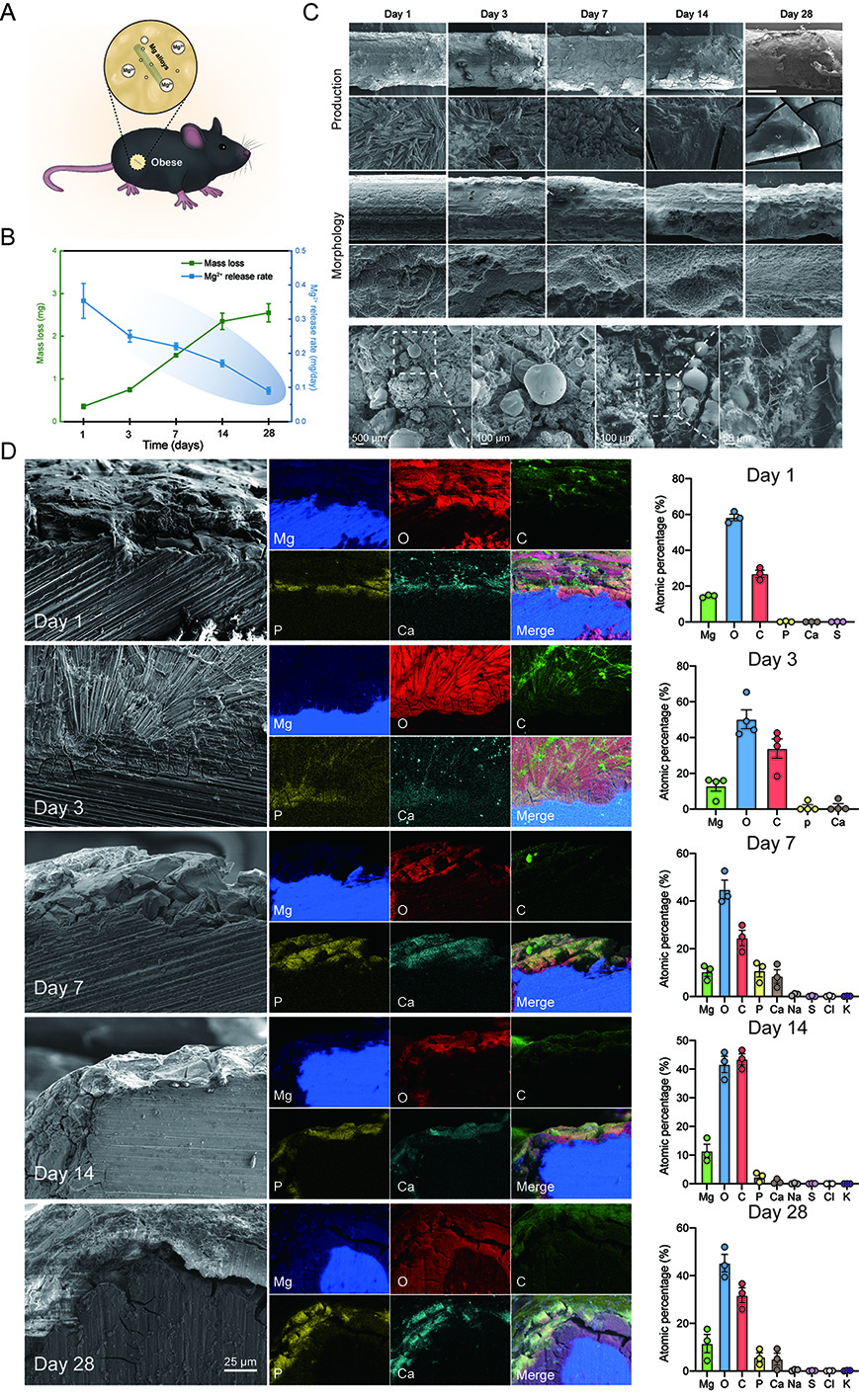


**Figure S25. The biodegradation process and related corrosion products of Mg wire implanted in adipose tissue.**

(A) A schematic diagram showing the biodegradable Mg wire as a potential implant device for weight loss;

(B) The relevance of implantation time with the mass loss rate and Mg release rate of Mg wires (n = 3);

(C) SEM images show the corrosion products and degradation morphology of Mg wires after implantation for 1, 3, 7, 14, and 28 days, respectively. Scar bar, 500 μm;

(D) Cross-sectional images of the *in vivo* corrosion products of the AMI during subcutaneous implantation and corresponding elemental mappings (n = 3).


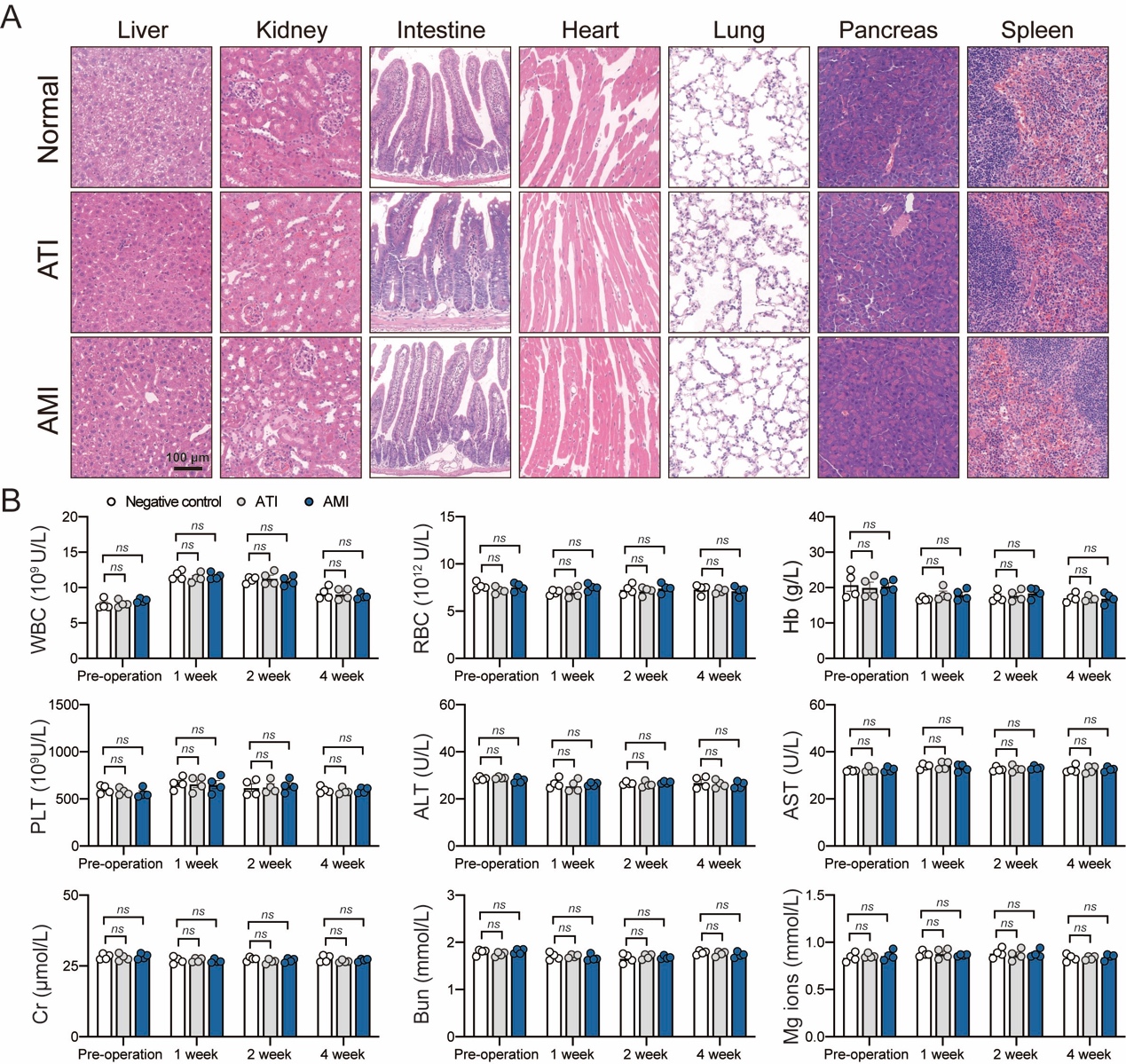


**Figure S26. Pathological images, element analysis, and blood biochemical analysis after AMI.**

**(**A and B) Pathological images of the internal organs (liver, kidney, intestine, heart, lung, pancreas, and spleen) (A) and Blood biochemical analysis of peripheral blood (B) before and after transplantation for 1, 2, and 4 weeks (n = 4).

These data are the mean ± SD. Data were expressed as means ± SEM. B was calculated by two-way ANOVA with Bonferroni’s multiple comparison test.


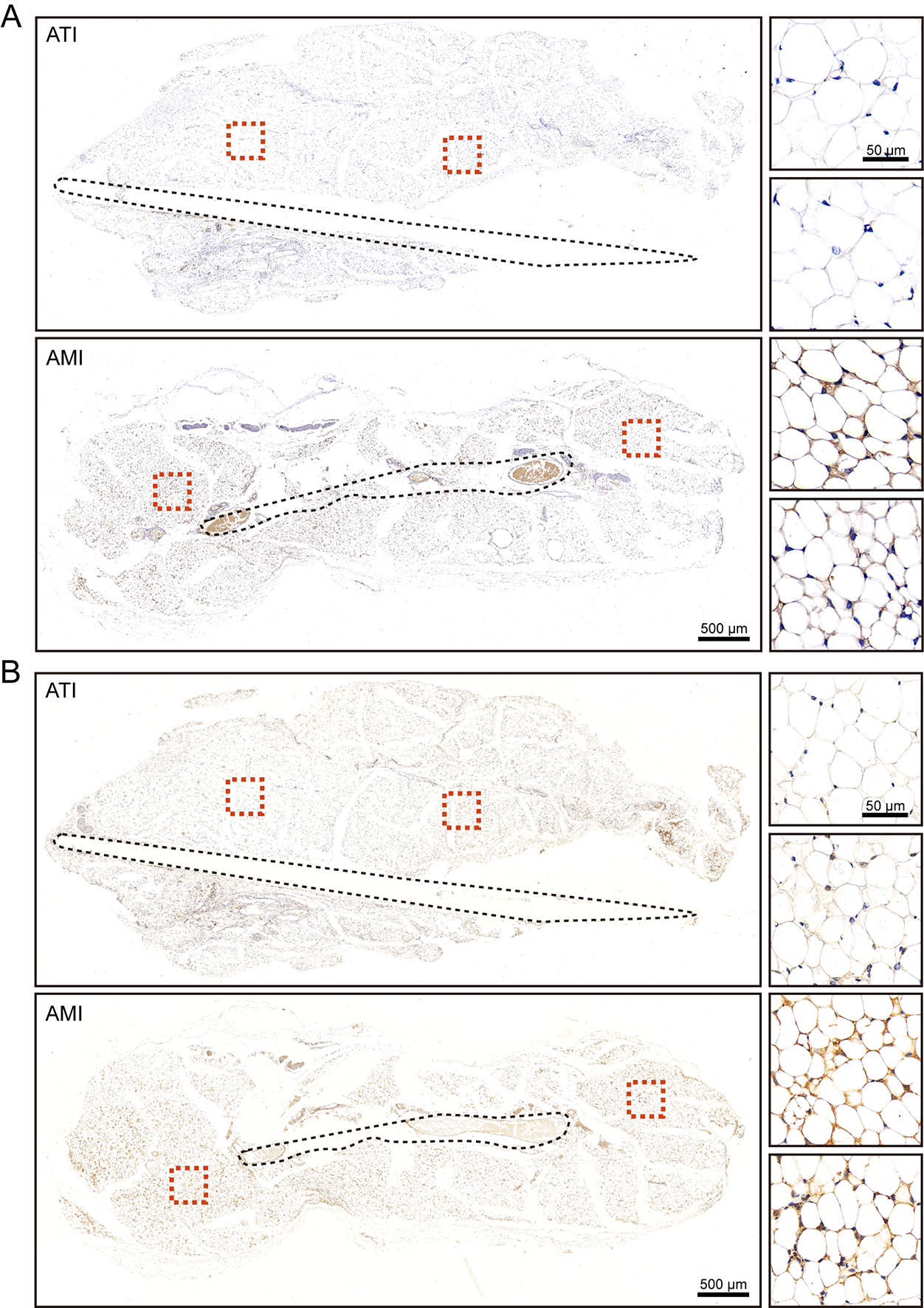


**Figure S27. M2 macrophage marker gene expression and UCP1 protein levels are increased in adipose tissue after AMI.**

Immunohistochemical staining of CD206 (A) and UCP1 (B) in the scWAT after AMI.

**
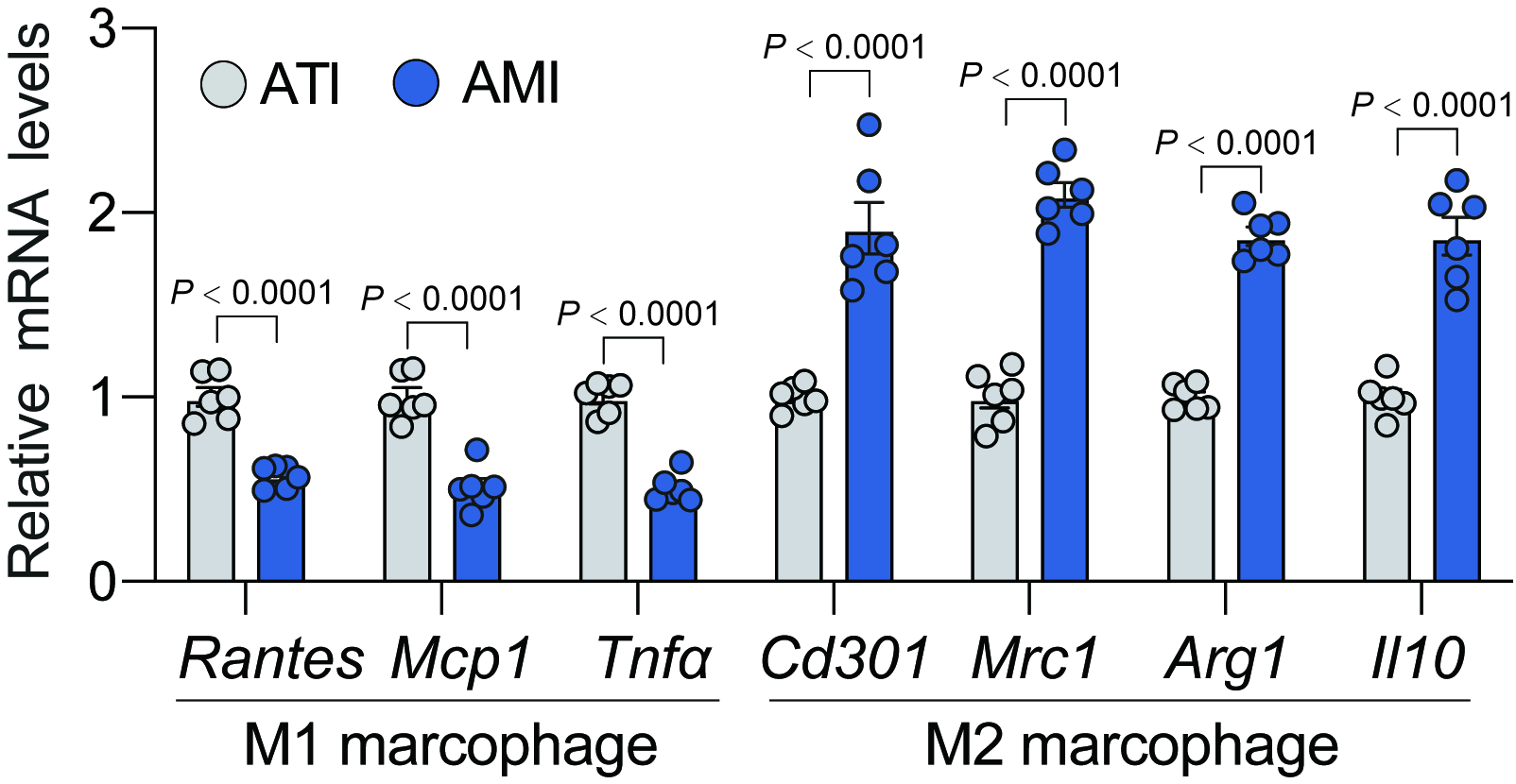
**

**Figure S28. AMI-released Mg promotes M2 macrophage polarization under normal chow diet .**

Representative M1 and M2 macrophage marker gene expression in scWAT after AMI (n = 6). Data were expressed as means ± SEM and were calculated by unpaired two-tailed Student’s t test.


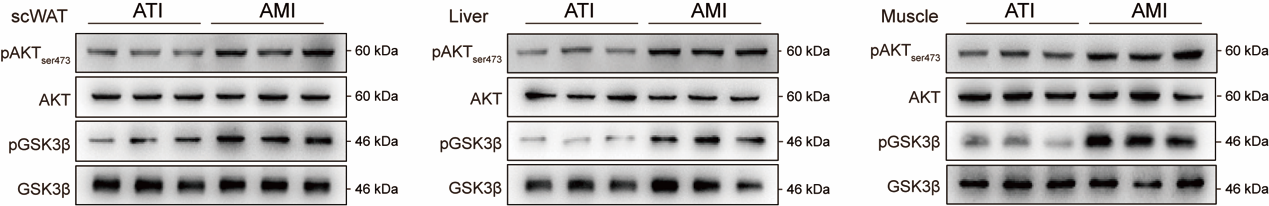


**Figure S29. Effects of AMI on insulin signaling.**

Representative immunoblots showing effects of AMI on insulin signaling in scWAT, liver, and muscle tissues.


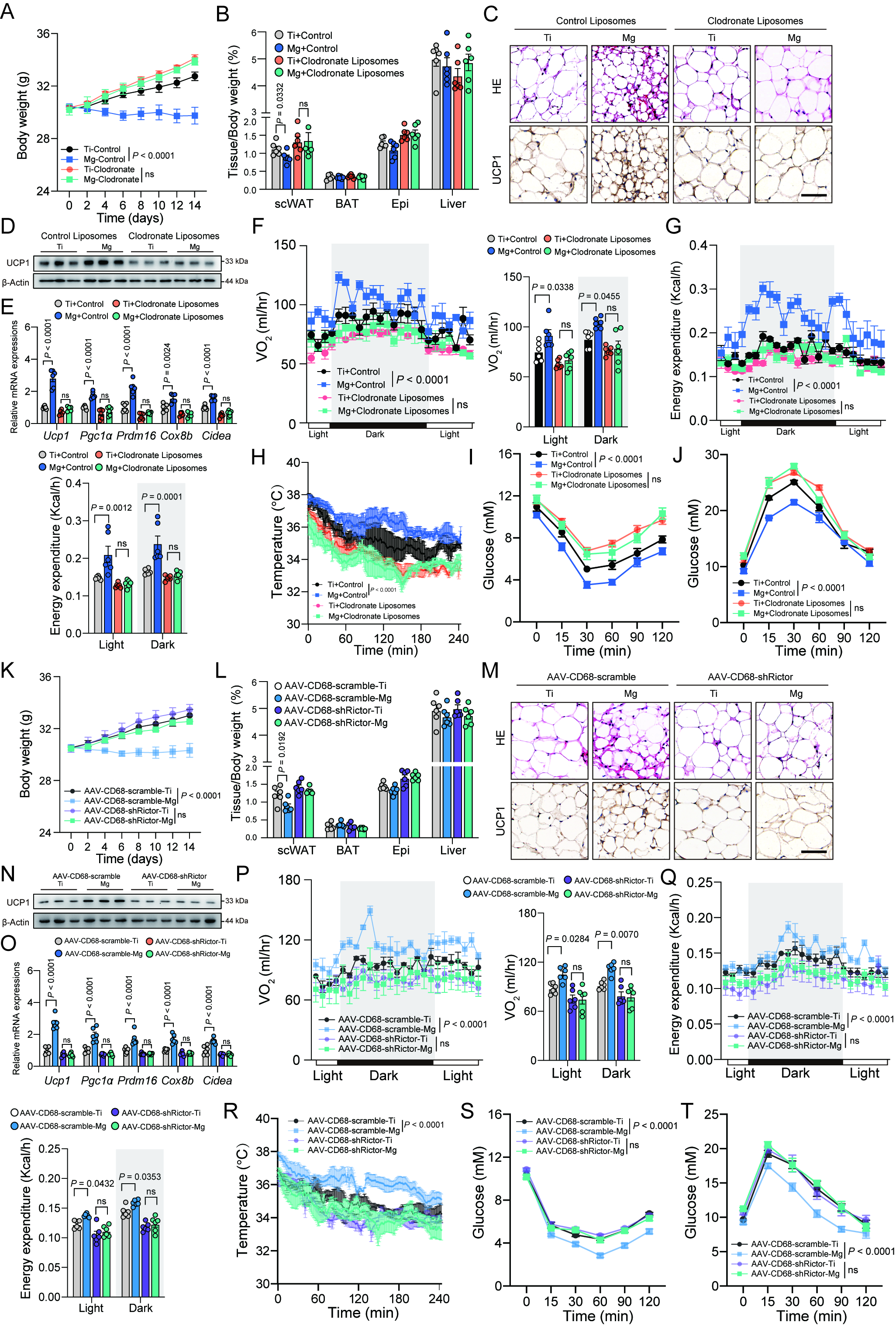


**Figure S30. AMI promotes thermogenesis and insulin sensitivity through M2 macrophages.**

(A-G) Body weight (A), indicated tissue weight (B), representative H&E staining (Top) and UCP1 immunohistochemical (Down) staining (C), UCP1 protein levels (D), representative thermogenic gene expression (E) in scWAT, VO_2_ (F) and energy expenditure (G) of AMI-transplanted HFD-fed mice with local injection of clodronate liposomes for 2 weeks (n = 4). Scale bar, 50 μm;

(H) Core temperature of AMI-transplanted HFD-fed mice with local injection of clodronate liposomes for 2 weeks under cold exposure for 4 h (n = 3);

(I and J) Glucose tolerance tests (I) and insulin tolerance tests (J) of AMI-transplanted HFD-fed mice with local injection of clodronate liposomes for 2 weeks (n = 6);

(K-Q) Body weight (K), indicated tissue weight (L), representative H&E staining (Top) and UCP1 immunohistochemical (Down) staining (M), UCP1 protein levels (N), representative thermogenic gene expression (O) in scWAT, VO_2_ (P) and energy expenditure (Q) of AMI-transplanted HFD-fed mice with local injection of AAV-CD68-shRicor to block mTORC2 activation in macrophages (n = 6);

(R) Core temperature of AMI-transplanted HFD-fed mice with local injection of AAV-CD68-shRicor under cold exposure for 4 h (n = 3). Scale bar, 50 μm;

(S and T) Glucose tolerance tests (S) and insulin tolerance tests (T) of AMI-transplanted HFD-fed mice with local injection of AAV-CD68-shRicor (n = 6).

Data were expressed as means ± SEM. A, B, E-L, and O-T were calculated by two-way ANOVA with Bonferroni’s multiple comparison test; F-H and P-R were analyzed by ANCOVA with body weight as covariant.


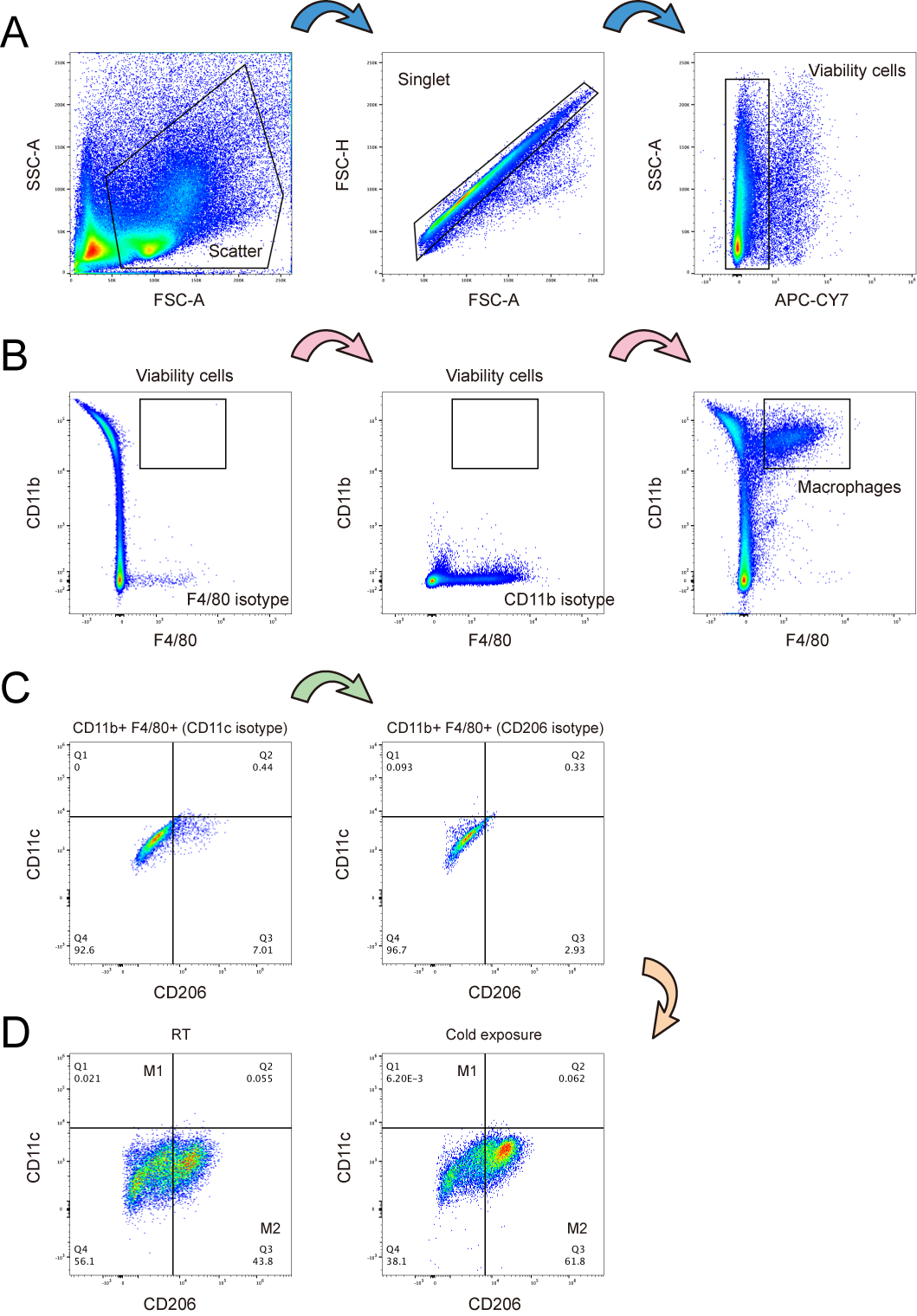


**Figure S31. The gating strategy for isolated macrophage from SVF.**

(A) Gating strategy to sort singlet and viability cells from mice under room temperature (25℃) or cold exposure (4℃);

(B) Modified FMO controls incorporating isotype antibodies for F4/80 and CD11b were used to align quadrant gates;

(C) Modified FMO controls incorporating isotype antibodies for F4/80^+^ CD11b^+^ following CD11c^+^ and F4/80^+^ CD11b^+^ following CD206^+^ were used to align quadrant gates;

(D) Based on A-C gating strategy, comparison of proportion of CD206^+^ (M2) or CD11c^+^ (M1) from the of F4/80^+^CD11b^+^ ATMs of mice under room temperature and cold exposure.

**Table S1. Clinical data of lean and obese individuals.**

ID 1-16 were lean individuals, and 17-32 were obese patients.

| ID | Age | Gender | BMI | Lean/Obesity | Glucose (mM) |
| --- | --- | --- | --- | --- | --- |
| 1 | 60 | Male | 21.26 | Lean | 4.7 |
| 2 | 52 | Female | 25.26 | Lean | 5.3 |
| 3 | 13 | Male | 23.15 | Lean | 5.3 |
| 4 | 62 | Female | 24.03 | Lean | 4.8 |
| 5 | 69 | Female | 23.92 | Lean | 5.9 |
| 6 | 63 | Male | 25.22 | Lean | 5.2 |
| 7 | 58 | Male | 21.04 | Lean | 5.7 |
| 8 | 32 | Female | 21.83 | Lean | 7.4 |
| 9 | 73 | Male | 16.46 | Lean | 5.1 |
| 10 | 56 | Male | 25.95 | Lean | 4.7 |
| 11 | 66 | Female | 18.55 | Lean | 5.2 |
| 12 | 18 | Female | 18.41 | Lean | 4.6 |
| 13 | 60 | Female | 22.43 | Lean | 5.1 |
| 14 | 46 | Female | 21.45 | Lean | 8.9 |
| 15 | 83 | Male | 23.57 | Lean | 4.9 |
| 16 | 66 | Male | 24.84 | Lean | 6.3 |
| 17 | 64 | Male | 55.24 | Obesity | 7.8 |
| 18 | 62 | Female | 37.32 | Obesity | 6.2 |
| 19 | 45 | Female | 38.82 | Obesity | 4.6 |
| 20 | 39 | Female | 53.55 | Obesity | 5.1 |
| 21 | 68 | Male | 29.98 | Obesity | 6.8 |
| 22 | 57 | Female | 41.29 | Obesity | 6.2 |
| 23 | 73 | Female | 39.18 | Obesity | 5.9 |
| 24 | 34 | Male | 44.23 | Obesity | 6.8 |
| 25 | 24 | Male | 40.35 | Obesity | 5.6 |
| 26 | 33 | Female | 36.85 | Obesity | 8.1 |
| 27 | 23 | Male | 34.34 | Obesity | 6.5 |
| 28 | 35 | Male | 35.16 | Obesity | 8.1 |
| 29 | 17 | Male | 34.79 | Obesity | 6.8 |
| 30 | 31 | Male | 56.60 | Obesity | 7 |
| 31 | 17 | Male | 31.91 | Obesity | 4.6 |
| 32 | 18 | Male | 35.81 | Obesity | 7.4 |

**Table S2. Primers for RT-PCR.**

| Genes | Forward primers 5’ to 3’ | Reversed primers 5’ to 3’ |
| --- | --- | --- |
| Mouse *Ucp1* | AGGCTTCCAGTACCATTAGGT | CTGAGTGAGGCAAAGCTGATTT |
| Mouse *Pgc1a* | TATGGAGTGACATAGAGTGTGCT | GTCGCTACACCACTTCAATCC |
| Mouse *Cidea* | TGACATTCATGGGATTGCAGAC | CATGGTTTGAAACTCGAAAAGGG |
| Mouse *Prdm16* | CCACCAGCGAGGACTTCAC | GGAGGACTCTCGTAGCTCGAA |
| Mouse *Cox8b* | TGTGGGGATCTCAGCCATAGT | AGTGGGCTAAGACCCATCCTG |
| Mouse *Rantes* | GCTGCTTTGCCTACCTCTCC | TCGAGTGACAAACACGACTGC |
| Mouse *Mcp1* | TAAAAACCTGGATCGGAACCAAA | GCATTAGCTTCAGATTTACGGGT |
| Mouse *Tnfα* | CAGGCGGTGCCTATGTCTC | CGATCACCCCGAAGTTCAGTAG |
| Mouse *Cd301* | CAATGTGGTTAGTTGGATCGGC | CCCAGTTCTTAAAGCCTTTCTCA |
| Mouse *Mrc1* | CTCTGTTCAGCTATTGGACGC | CGGAATTTCTGGGATTCAGCTTC |
| Mouse *Arg1* | CTCCAAGCCAAAGTCCTTAGAG | AGGAGCTGTCATTAGGGACATC |
| Mouse Il*10* | CTTACTGACTGGCATGAGGATCA | GCAGCTCTAGGAGCATGTGG |
| Mouse *Mrs2* | GGTGATGTGCTCCGGTTTAGA | TGGCCTGGAGTGCTAACTCAT |
| Mouse *Cnnm3* | GACTCCGGCACTGTCCTAGA | AGTGGATGGTTGTAGAAGCGG |
| Mouse *Cnnm4* | CTGCACATCCTTCTCGTTATGG | TGCGAGCATACTTTCTCTCCTT |
| Mouse *Slc41a1* | CCACCAGCCGAATGGTACTG | TGACATCGTCACTCTCGTTACT |
| Mouse *Slc41a2* | GCAGCCCCAAGTACGATGAC | CAGCAGAAACCGTTCCAAACC |
| Mouse *Magt1* | GAGAGAAGCGTGAACATGGC | TTATTGGCCCATTCCATCAGC |
| Mouse *Trpm6* | AAAATCGGATCATCTGTTGCACT | AATGGGCTTTTAGGGCATCCC |
| Mouse *Trpm7* | AGGATGTCAGATTTGTCAGCAAC | CCTGGTTAAAGTGTTCACCCAA |
| Mouse *Actb* | GTGACGTTGACATCCGTAAAGA | GCCGGACTCATCGTACTCC |
| Mouse *Gapdh* | CAAGGTCATCCATGACAACTTTG | GGCCATCCACAGTCTTCTGG |
| Human *Mrs2* | ACTTAGAGCAATGGCTGTTCC | GCATCCAAGGTCTCAAGGATCAG |
| Human *Cnnm3* | CATCCCGGTGTACGAGGAG | GGTTGTAGAAACGAGTGATGGT |
| Human *Cnnm4* | GCGAGAGCATGAAGCTGTATG | AGCAGTGAGTCCTTGTCCGT |
| Human *Slc41a1* | CCAGAGCAACGAAAGTGACGA | TACTTGCAGCCCGATGGAAAA |
| Human *Slc41a2* | AATAATGGGCACGCATCATCA | AAGTCTCCCTTCCATCACAGTAA |
| Human *Magt1* | ACCTAGCCGGAGCAAAGTTTC | CGTCGCAAACGATGAGCAG |
| Human *Trpm6* | AGCACAATCATACCCAGCTC | CATGGTCTCCAATCAGTCGGC |
| Human *Trpm7* | ACTGGAGGAGTAAACACAGGT | TGGAGCTATTCCGATAGTGCAA |
| Human *CD68* | GGAAATGCCACGGTTCATCCA | TGGGGTTCAGTACAGAGATGC |
| Human *RANTES* | CCAGCAGTCGTCTTTGTCAC | CTCTGGGTTGGCACACACTT |
| Human *TNFα* | CCTCTCTCTAATCAGCCCTCTG | GAGGACCTGGGAGTAGATGAG |
| Human *MCP1* | CAGCCAGATGCAATCAATGCC | TGGAATCCTGAACCCACTTCT |
| Human *CD301* | AGCAACTTCACCTCAAACACTG | AGATGCTATCGTTTCTTCCAAGC |
| Human *MRC1* | TCCGGGTGCTGTTCTCCTA | CCAGTCTGTTTTTGATGGCACT |
| Human *ARG1* | GTGGAAACTTGCATGGACAAC | AATCCTGGCACATCGGGAATC |
| Human *IL10* | GACTTTAAGGGTTACCTGGGTTG | TCACATGCGCCTTGATGTCTG |
| Human *ACTB* | CATGTACGTTGCTATCCAGGC | CTCCTTAATGTCACGCACGAT |
